# *Schistosoma japonicum* IAP and Teg20 safeguard tegumental integrity by inhibiting cellular apoptosis

**DOI:** 10.1371/journal.pntd.0006654

**Published:** 2018-07-25

**Authors:** Juntao Liu, Bikash R. Giri, Yongjun Chen, Rong Luo, Tianqi Xia, Christoph G. Grevelding, Guofeng Cheng

**Affiliations:** 1 Department of Animal Schistosomiasis, Shanghai Veterinary Research Institute, Chinese Academy of Agricultural Sciences, Key Laboratory of Animal Parasitology, Ministry of Agriculture, Shanghai, China; 2 Institute for Parasitology, BFS, Justus-Liebig-University, Giessen, Germany; Queen's University Belfast, UNITED KINGDOM

## Abstract

Schistosomes are causative agents of human schistosomiasis, which is endemic in tropical and subtropical areas of the world. Adult schistosomes can survive in their final hosts for several decades, and they have evolved various strategies to overcome the host immune response. Consequently, understanding the mechanisms that regulate parasitic cell survival will open avenues for developing novel strategies against schistosomiasis. Our previous study suggested that an inhibitor of apoptosis protein in *Schistosoma japonicum* (SjIAP) may play important roles in parasitic survival and development. Here, we demonstrated that SjIAP can negatively regulate cellular apoptosis in *S*. *japonicum* by suppressing caspase activity. Immunohistochemistry analysis indicated that SjIAP ubiquitously expressed within the worm body including the tegument. Silencing of *SjIAP* expression via small interfering RNA led to destruction of the tegument integrity in schistosomes. We further used co-immunoprecipitation to identify interaction partners of SjIAP and revealed the tegument protein SjTeg-20 as a putative interacting partner of SjIAP. The interaction between SjIAP and SjTeg-20 was confirmed by a yeast two-hybrid (Y2H) assay. Moreover, results of a TUNEL assay, RNA interference, scanning and transmission electron microscopy, caspase assays, transcript profiling, and protein localization of both interacting molecules provided first evidence for an essential role of SjIAP and SjTeg-20 to maintain the structural integrity of the tegument by negatively regulating apoptosis. Taken together, our findings suggest that the cooperative activities of SjIAP and SjTeg-20 belong to the strategic inventory of *S*. *japonicum* ensuring survival in the hostile environment within the vasculature of the final host.

## Author summary

Schistosomiasis is a worldwide public health concern particularly in developing countries. The causative agents, schistosomes, can survive within the vascular system of their final hosts for several decades despite facing the host’s immune response. Therefore, elucidating the mechanism of cell survival will contribute to the understanding of host-parasite interaction and may lead to the identification of suitable targets for developing novel strategies against schistosomiasis. Inhibitor of apoptosis proteins are highly conserved proteins functioning as endogenous inhibitors of apoptotic cell death. Here, we demonstrated that an inhibitor of apoptosis protein of *Schistosoma japonicum* (SjIAP) governs the integrity of the tegument of schistosomes by inhibiting cellular apoptosis of the parasite. Further studies revealed that SjTeg-20, an *S*. *japonicum* tegumental protein, cooperates with SjIAP to inhibit apoptosis in schistosomes. Our findings provide new insights into the role of SjIAP and SjTeg-20 in maintaining the integrity of the worm tegument by negatively regulating apoptosis.

## Introduction

As one of the neglected tropical diseases, schistosomiasis is an important public health concern affecting more than 200 million people in 78 tropical and subtropical countries [1]. Schistosomiasis is caused by parasites of the genus of *Schistosoma*. Although schistosomes cannot proliferate within their final hosts, the main pathology is due to the large number of eggs deposited that become trapped in the host liver and other organs. Mature female worms can produce hundreds (e.g. *S*. *mansoni*, *S*. *haematobium*) to thousands (e.g. *S*. *japonicum*) of eggs per day. Importantly, paired adult schistosomes can survive in the final host for several decades. This longevity along with the production of a large number of eggs causes the pathologic consequences and contributes to disease spreading. Uncovering the parasite’s principles of survival in an otherwise aggressive host environment will provide valuable insights into parasite-host interaction which may help to find novel intervention strategies against schistosomiasis. These are urgently needed in face of the facts that there is no vaccine available and only one drug, praziquantel, which is widely used to fight the disease [2].

Apoptosis is a distinct form of programmed cell death and plays a significant role in tissue homeostasis during development [3–5]. During pathogen infection, parasites can manipulate host apoptotic pathways to prevent apoptosis of infected host cells [6, 7]. In addition, molecules secreted form schistosome eggs induce pro-apoptotic effects against skin T-lymphocytes and liver cells [8, 9]. Indeed, apoptosis has been proposed to play an important role in schistosome survival within the host [10]. In this context, transcripts of several orthologs of caspases have been identified in different life stages of schistosomes [11, 12], and genomic studies revealed that schistosomes have an active apoptotic pathway [11, 13–15].

Opposite to factors triggering apoptosis, other factors also negatively regulate apoptosis including inhibitors of apoptosis proteins (IAPs) and B cell lymphoma 2 (Bcl-2). In particular, Bcl-2 negatively regulates apoptosis in schistosomes [15]. For example, Lee et al documented a Bcl-2-regulated apoptosis pathway in *S*. *japonicum* and *S*. *mansoni* and demonstrated target of Bcl-2 prosurvival proteins may have a potential against schistosomiasis [15]. IAPs represent a highly conserved protein family supporting pro-survival signaling pathways through preventing the effector phase of apoptosis. IAPs were first reported in baculoviruses as potent inhibitors of apoptosis in infected insect cells, and several IAP orthologs sharing structural features have since been identified in a variety of species including yeast, nematodes, cestodes, trematodes, insects, fishes, and mammals [16]. Originally, IAPs were shown to inhibit cell apoptosis during viral infection, which could halt viral replication by delaying RNA transcript and protein translation [17]. Recent studies have further expanded on the functions of IAPs, which can be involved in the regulation of many biological processes such as cell division, morphogenesis, cell cycle, heavy metal homeostasis, NF-kB activation, immune response, and mitogen-activated protein kinase signal transduction pathways [18–20].

IAPs usually contain one to three conserved protein motifs, namely the baculovirus IAP repeat (BIR), a RING finger domain, and a zinc-binding motif conferring E3-ubiquitin ligase activity [21, 22]. Previously, we conducted a genome-wide analysis of *S*. *japonicum* and found three apoptosis inhibitor homologs including SjIAP [23], SjCIAP [24], and SjBIRP [25]. Gene cloning and further sequence analysis indicated that both SjIAP and SjBIRP contain a BIR domain. Among these, SjIAP showed a markedly increased expression in the life-cycle stages associated with the final hosts [23]. This suggests that SjIAP may contribute to the parasite’s survival strategies within the final hosts. In the present study, we aimed to determine the mechanism by which SjIAP exerts its regulation function through investigating its roles in apoptosis. We determined the expression patterns of SjIAP throughout different developmental stages of the parasite and conducted immunohistochemistry to localize the protein. Furthermore, by RNA interference (RNAi) SjIAP was silenced to examine its specific effect on apoptosis. By co-immunoprecipitation and yeast-two-hybrid (Y2H) analysis we finally identified an interacting partner of SjIAP that cooperates in apoptosis regulation and evaluated the effects of this interaction *in vivo* using a mouse infection model.

## Results

### SjIAP is expressed in different life stages of *S*. *japonicum* and localizes in the worm tegument of adults

**Fig 1:**
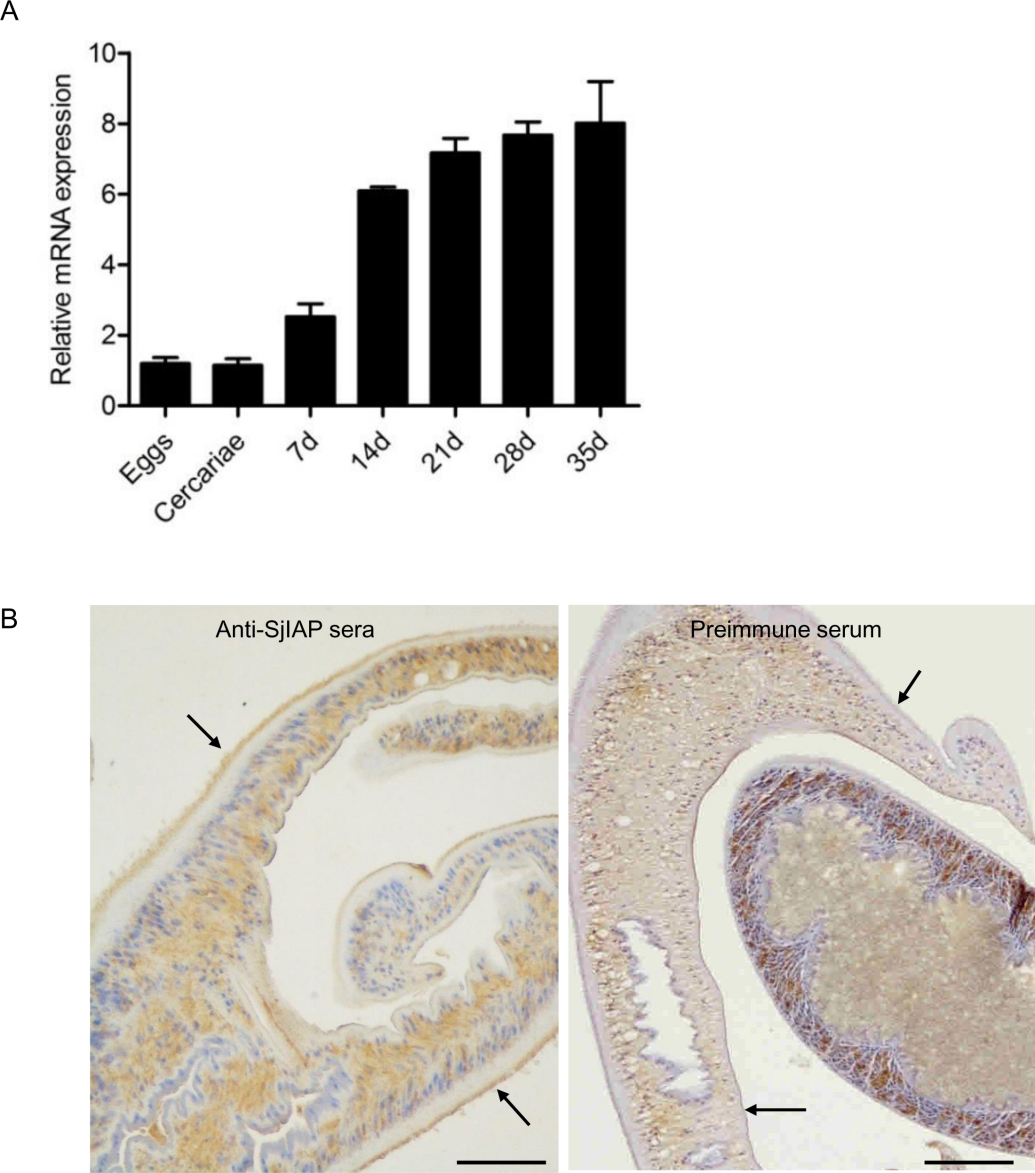
Developmental expression and localization of SjIAP in *S*. *japonicum*. (A) Expression of *SjIAP* at different stages of the *S*. *japonicum* life cycles. Eggs were isolated from liver of rabbits infected with *S*. *japonicum* cercariae, cercariae were collected from infected *Oncomelania hupensis* snails, and the parasites were collected from infected rabbits at 7, 14, 21, 28, and 35 days post-infection, respectively. The mRNA expression levels of *SjIAP* relative to *SjNADH* were analyzed by qRT-PCR. Data illustrate representative results and show the mean and standard errors derived from triplicate experiments. (B) Immunohistochemistry analysis of SjIAP in adult schistosomes using an antiserum against SjIAP [21]. Arrows indicate the schistosome tegument. Scale bars: 50 μm.

Previously, we found that the expression of SjIAP significantly increased during the development of the adult stages in the final host [23]. To further corroborate this result, we expanded the analysis to determine the SjIAP mRNA expression levels of seven different life stages relative to the control gene *S*. *japonicum nicotinamide adenine dinucleotide dehydrogenase* (*SjNADH*) [26, 27] by qRT-PCR. As shown in Fig 1A, *SjIAP* mRNAs were detected in all studied stages of the schistosome life cycle with significantly higher levels identified in the stages associated with the final host. Moreover, immunohistochemistry were performed to determine the localization of SjIAP using our previously generated anti-SjIAP antibody [23]. The results indicated that SjIAP is concentrated in the tegument of adult parasites but also ubiquitously expressed within the worm body (Fig 1B).

### SjIAP silencing resulted in increased caspase activity and worm mortality

**Fig 2:**
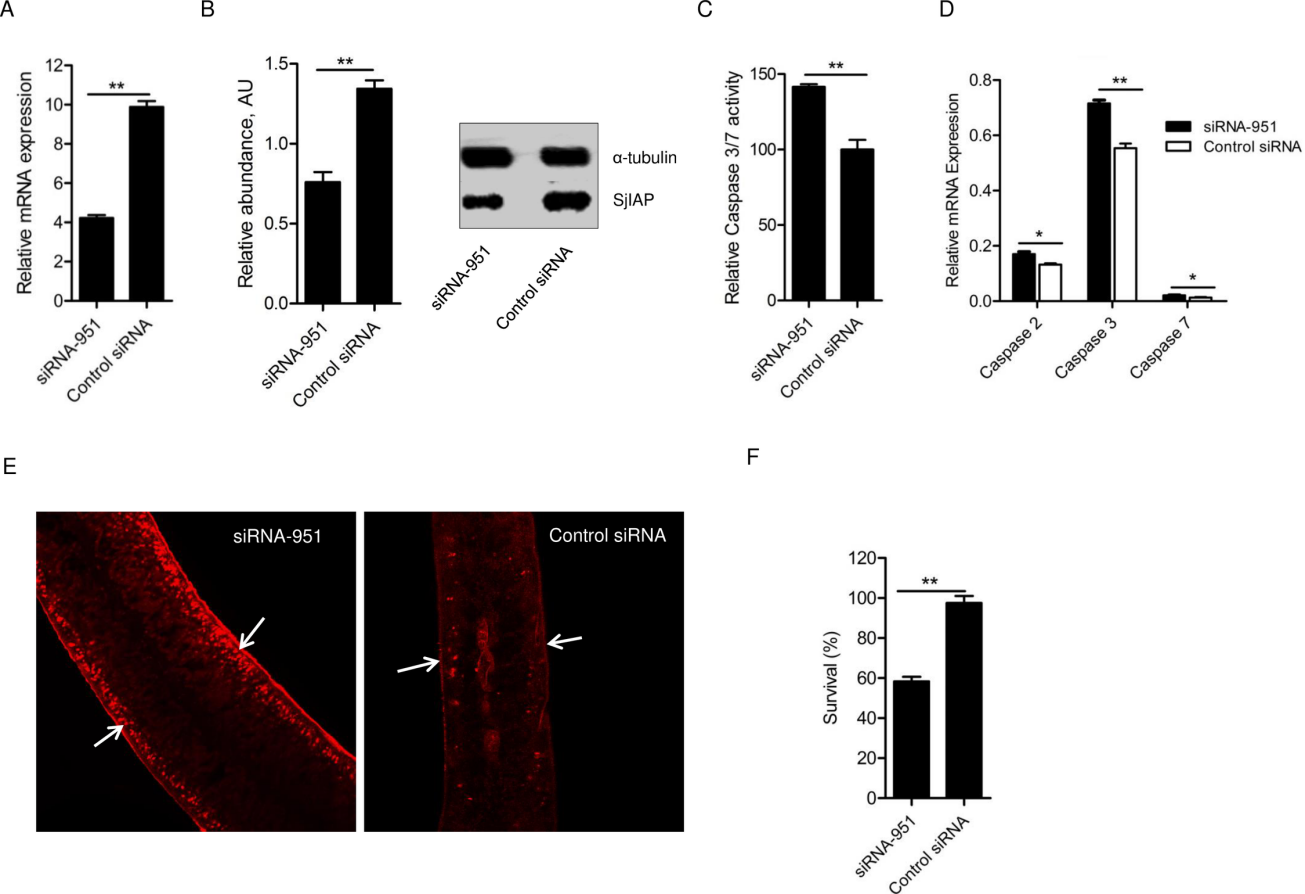
Effect of SjIAP silencing on caspase activity and worm survival. (A) Incubation of adult worms with *SjIAP* siRNA-951 *in vitro* led to a significant decrease of *SjIAP* expression at the transcript level. At 4 days of post-treatment, the RNA was isolated from the worms. Transcript levels of *SjIAP* relative to the reference gene *SjNADH* were determined in parasites transfected with *SjIAP* siRNA and control siRNA using qRT-PCR. The data illustrate representative results and show mean±SEM derived from triplicate experiments. ** *P* ≤ 0.01. (B) Western blot analysis of the effect of SjIAP silencing at the protein level. The densitometry results (arbitrary units [AU]) of the Western blot analyzed by Image J were shown as a bar graph. Each bar represents mean±SEM from triplicate experiments. Representative Western blot result was also shown. ** *P* ≤ 0.01. (C) SjIAP silencing resulted in increased caspase 3/7 activity. At 4 days of post-treatment, the protein lysates of worms were prepared and used to determine caspase 3/7 activity. Data are representative result and shown the mean and standard errors from triplicate experiments. ** *P* ≤ 0.01. (D) qRT-PCR analysis of the transcript levels of different caspases (caspase 2, caspase 3, and caspase 7) in SjIAP-silenced and control schistosomes determined relative to *SjNADH* at 4 days post-treatment. (E) SjIAP silencing led to an increased number of TUNEL^+^ cells in the tegument (arrows) of schistosomes at 4 days post-treatment. Data are representative results from at least 20 worms investigated in at least three independent experiments. (F) SjIAP silencing resulted in increased worm mortality. At 4 days post-treatment, the worms were stained with Hoechst 33258 dye, and the mortality was determined by microscopy. Data are representative and show the mean and standard errors from three separate experiments. ** *P* ≤ 0.01.

In a previous study, we found that recombinant SjIAP significantly inhibited in caspase 3/7 activity in adult worm lysates [23], which suggested its involvement in apoptosis regulation of schistosomes. To gain further insights into the specific functions of SjIAP, we designed three small interfering RNA (siRNA) duplexes targeting *SjIAP* mRNA, which were electroporated into adult schistosomes cultured *in vitro*. The effect of siRNA silencing on the mRNA and protein expression of SjIAP was evaluated by qRT-PCR and western blot, respectively. Finally, we determined that the siRNA-951 duplex exerted the best effect in silencing SjIAP expression [28]. Therefore, in the present study, we delivered this siRNA (siRNA-951) duplex into adult worms and determined the effect of SjIAP silencing on caspase activity and worm mortality, respectively. Four days post-treatment, the qRT-PCR results indicated a 67.5% (p = 0.00007) reduction of the transcript level of *SjIAP* compared to that of parasites treated with control siRNA (Fig 2A). Western blot analysis further validated this reduction at the protein level (Fig 2B). Notably, the caspase activity assay demonstrated that schistosomes treated with *SjIAP* siRNA had significantly increased caspase 3/7 activity compared with that of control parasites (Fig 2C). Because of the lack of specific antibodies against active caspases for *S*. *japonicum*, we also used qRT-PCR to determine the transcript levels of three *S*. *japonicum* caspases including caspase 2, caspase 3, and caspase 7, which all had significantly increased transcripts levels in SjIAP-silenced worms (Fig 2D). Moreover, a whole-mount assay was used to examine terminal deoxynucleotidyl transferase dUTP nick-end labeling (TUNEL), which is a common method to detect double-stranded breaks in the DNA of cells undergoing programmed cell death. The SjIAP-silenced parasites exhibited large clusters of TUNEL^+^ cells, mainly present in the tegument although unspecific staining was observed at outer tegumental syncytium (Fig 2E). This indicates that the relief of inhibition by SjIAP could accelerate the process of apoptosis. Furthermore, siRNA treatment led to a clear decrease in worm survival. Over an observation period of 4 days, about 41% of the worms died in culture (Fig 2F).

### SjIAP inhibition led to morphological alteration in the tegument of *S*. *japonicum*

**Fig 3:**
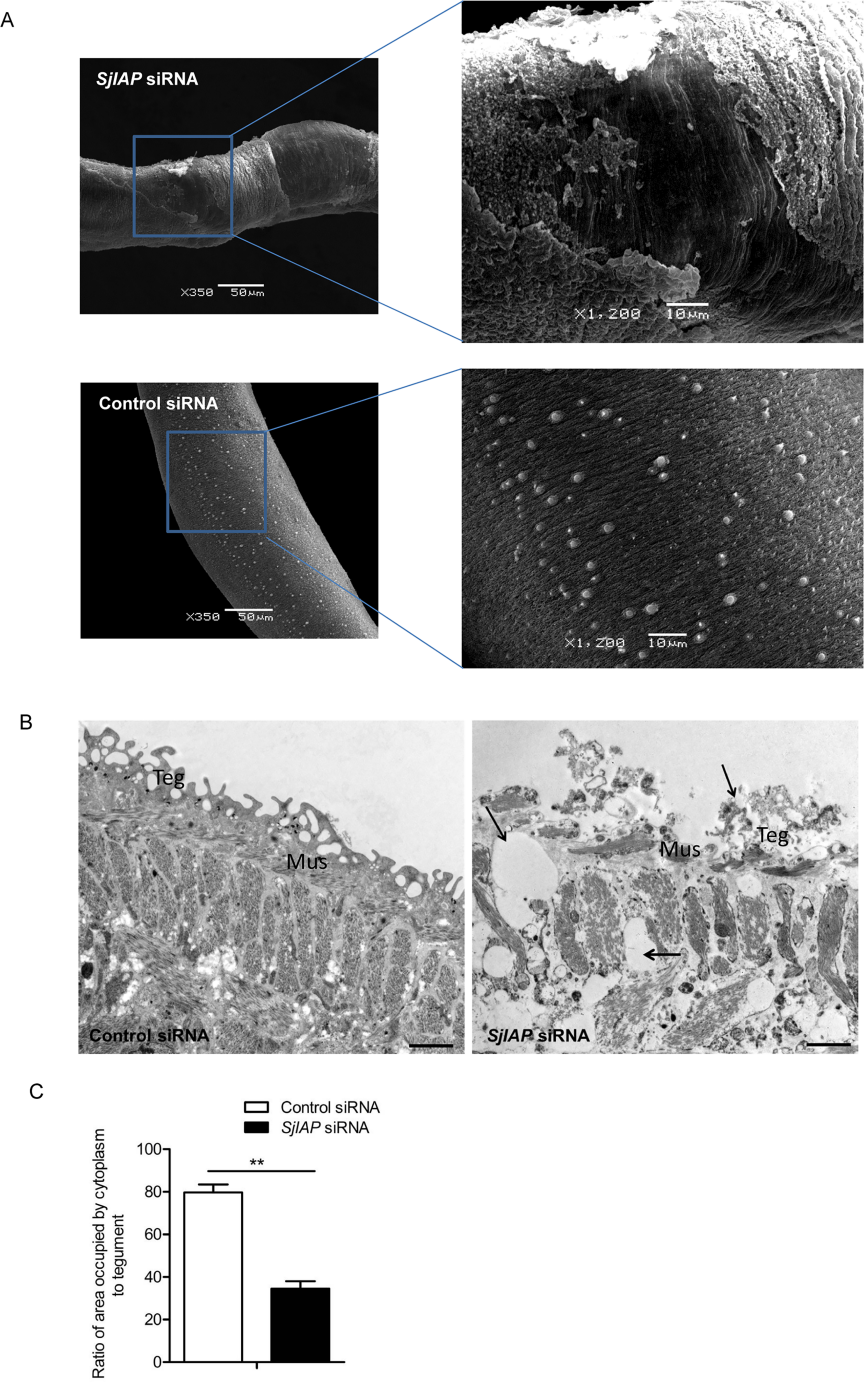
SjIAP inhibition by siRNA led to morphological alterations in the tegument of *S*. *japonicum*. (A) Scanning electronic microscopy (SEM) of the tegument of *S*. *japonicum* following treatment with *SjIAP* siRNA. Tegument ridges and sensory structures were observed in the mid part of the worm body. Data are representative results from at least 20 worms investigated in three independent experiments. (B) Transmission electronic microscopy (TEM) of the tegument of *S*. *japonicum* following treatment with *SjIAP* siRNA. Data are representative results from at least 15 worms investigated in three independent experiments. Scale bars: 2 μM. (C) Quantification of tegument changes due to SjIAP silencing shown in (B). Tegument defects were analyzed using Image J software based on the ratio of the area occupied by the tegument cytoplasm to the total area of the tegument. Data show the mean and standard errors derived from four randomly selected parasites. ** *P* ≤ 0.01 (Student’s t test, *SjIAP* siRNA vs control siRNA).

Since SjIAP localized in the tegument of schistosomes and elsewhere and SjIAP silencing resulted in increased worm mortality, we used scanning electron microscopy (SEM) to investigate morphological alterations of the schistosome surface area following SjIAP inhibition by siRNA. To this end, worms were harvested from mice at 22 days post-infection and then were electroporated either with *SjIAP* siRNA or control siRNA *in vitro*. At 4 days of post treatment, the surviving female worms were analyzed by SEM. In general, different regions of the schistosome tegument have particular features, including ridges, spines, ciliated hemispherical papillae, and sensory structures [29, 30]. We detected swollen ridges along with detachment and crumbling of the tegument in schistosomes treated with *SjIAP* siRNA while such alterations were not observed in the control worms (Fig 3A). These findings indicate that SjIAP silencing led to destruction of the integrity of the tegument. Similar results were also observed in male schistosomes treated with *SjIAP* siRNA. To further substantiate these observations, we examined the ultrastructure of the schistosome tegument in parasites treated with *SjIAP* siRNA using transmission electron microscopy (TEM). The obtained results revealed severe defects of the tegument architecture in the majority (82%) of treated worms. This included the formation of enlarged vacuoles along with damaged apical and syncytial cytoplasm in the tegument (Fig 3B). The apical cytoplasm of schistosoma tegument is the interface with hosts while the syncytial cytoplasm is localized on bands of musculature and is supported by tegumentary cell bodies. In addition, the musculature of SjIAP-silenced worms was more vacuolated as compared to that of control worms (Fig 3B). Examination of the ratio of the areas occupied by tegument cytoplasm to the total area of the tegument demonstrated that SjIAP silencing led to a significant reduction of the area occupied by the tegument cytoplasm (Fig 3C); no such alterations were found in the tegument of worms treated with control siRNA.

### Co-immunoprecipitation identified SjTeg-20 as potential interaction partner of SjIAP

**Fig 4:**
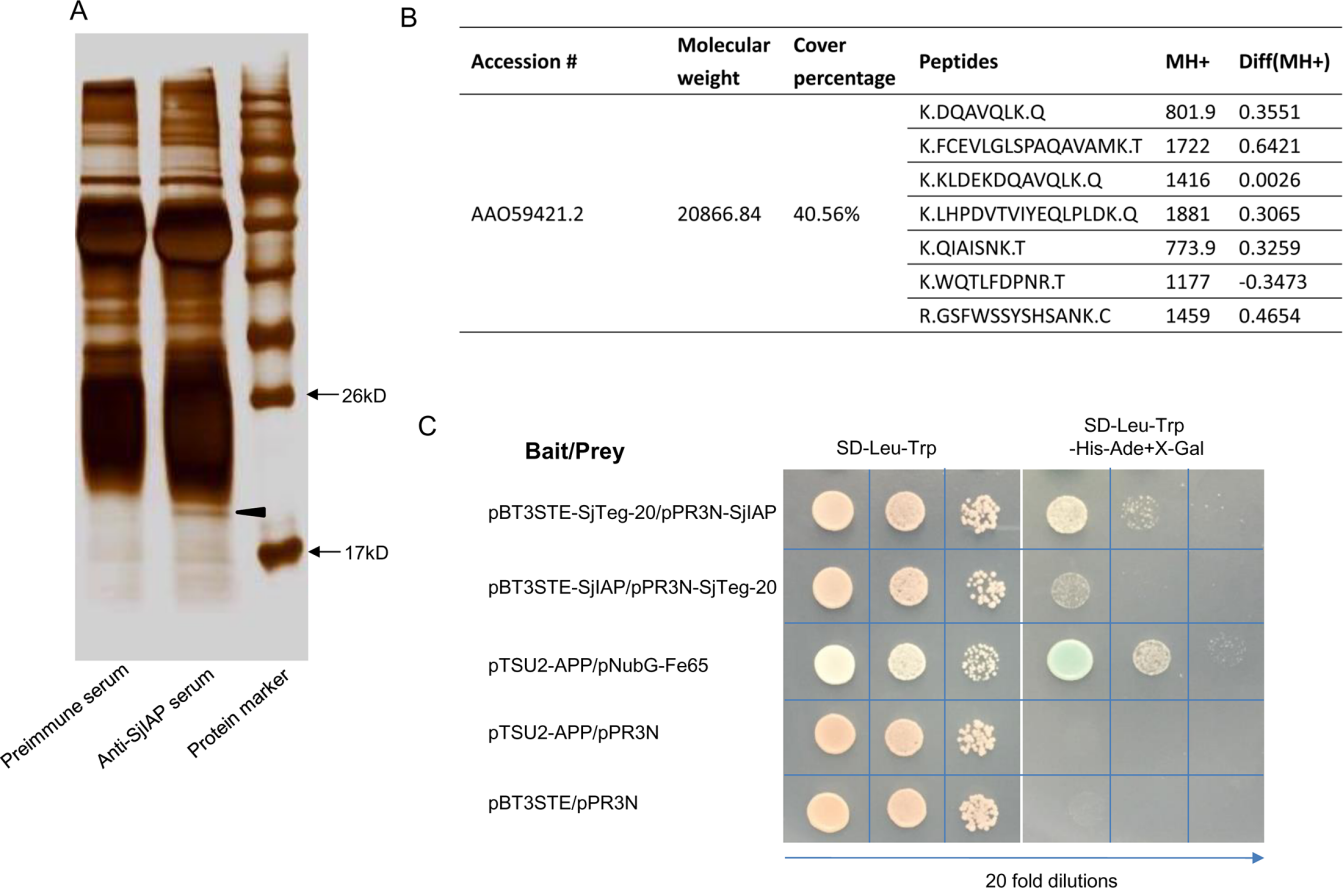
Identification and validation of SjTeg-20 as interaction partner of SjIAP. (A) For co-immunoprecipitation, protein lysates from adult schistosomes were incubated with anti-SjIAP serum and pre-immune serum. The pull-down products were separated by SDS-PAGE and visualized by sliver staining. (B) Search results for MS identification of the co-immunoprecipitation products. (C) Validation of the interaction between SjIAP and SjTeg-20 by Y2H analysis. All diploid strains in which the density was equalized before plating grew equally well under selection for both the bait and prey plasmids (SD-Leu-Trp). Twenty-fold dilutions for each diploid were inoculated. Prey SjIAP and bait SjTeg-20 strongly interacted on the reporter plate (SD-Leu-Trp-Ade-His+ X-gal). Bait SjIAP and prey SjTeg-20 also interacted but weaker as the positive control (pTSU2-APP+pNubG-Fe65) on the reporter plate. No interaction was detected with the negative control and empty plasmids.

To further unravel processes controlled by SjIAP to maintain tegument integrity, we carried out co-immunoprecipitation assays. To this end polyclonal SjIAP antibodies were used to isolate potential SjIAP interaction partners from protein lysates of adult worms. The pull-down products were separated by sodium dodecyl sulfate-polyacrylamide gel electrophoresis (SDS-PAGE) and then silver-stained (Fig 4A). An area of differing proteins was excised (Fig 4A and S1 Fig). Mass spectrometry (MS) analysis demonstrated that the specific band pulled-down by SjIAP antibodies corresponded to SjTeg-20 (AAO59421.2) (Fig 4B). We then used the DUAL membrane Y2H system to further validate the interaction between SjIAP and SjTeg-20. Briefly, SjIAP and SjTeg-20 were cloned into bait and prey plasmids, respectively, and the recombinant plasmids were co-transformed into NMY32 yeast cells, which were spread onto a selective plate (SD-Leu-Trp). The growing cells were inoculated onto the SD-TLHA+X-Gal plates (SD-Leu-Trp-Ade-His+X-Gal) with different dilutions. As shown in Fig 4C, after transformation of the prey plasmids encoding SjTeg-20 with the bait constructs containing SjIAP, yeast clones survived (Trp2/Leu2/Ade2/His2), indicating that SjIAP interacts with SjTeg-20 in yeast.

### Transcript profile and localization of SjTeg-20 corresponded to that of SjIAP in *S*. *japonicum*

**Fig 5:**
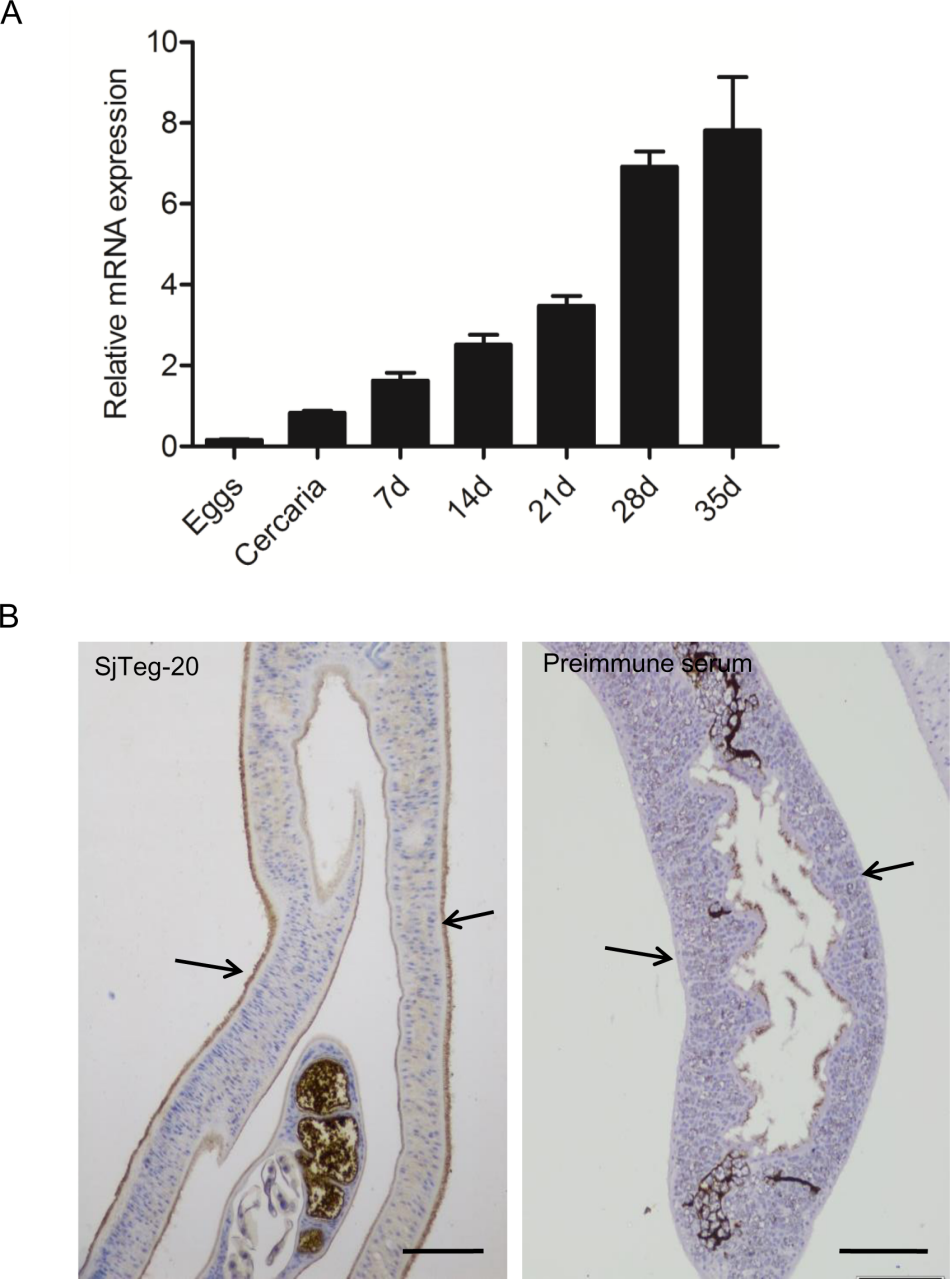
Expression profile of SjTeg-20 and its localization in *S*. *japonicum*. (A) qRT-PCR analysis of the expression profiles of *SjTeg-20* at different stages of *S*. *japonicum* relative to *SjNADH*. Data illustrate representative results and show the mean and standard errors derived from triplicate experiments. (B) Immunohistochemistry analysis of SjTeg-20 expression in the tegument (arrows) of adult schistosomes. Scale bars: 50 μm.

For characterizing SjTeg-20, we performed qRT-PCR to determine its transcript profile in different life-cycle stages. As shown in Fig 5A, transcript levels of *SjTeg-20* were mainly associated with adult stages. This finding corresponds to the results obtained for *SjIAP*. We next intended to immunolocalize SjTeg-20. Thus, the full-length *SjTeg-20* cDNA of adult worm was cloned into the bacterial expression vector pET28a (S2A Fig). Recombinant SjTeg-20 was successfully expressed and then purified by affinity chromatography (S2B and S2C Fig). New Zealand rabbits were immunized with the purified protein to generate anti-serum. Western blot analysis showed that the raised polyclonal antibodies were monospecific for recombinant SjTeg-20 (S2D Fig). Next the anti-serum was used to perform immunohistochemistry. The results indicated that SjTeg-20 is localized at the tegument of adult *S*. *japonicum* (Fig 5B). The corresponding transcript profiles and co-localization in the tegument strengthen the view that SjTeg-20 and SjIAP interact.

### SjTeg-20 silencing increased caspase activity and induced parasite mortality and tegument destruction

**Fig 6:**
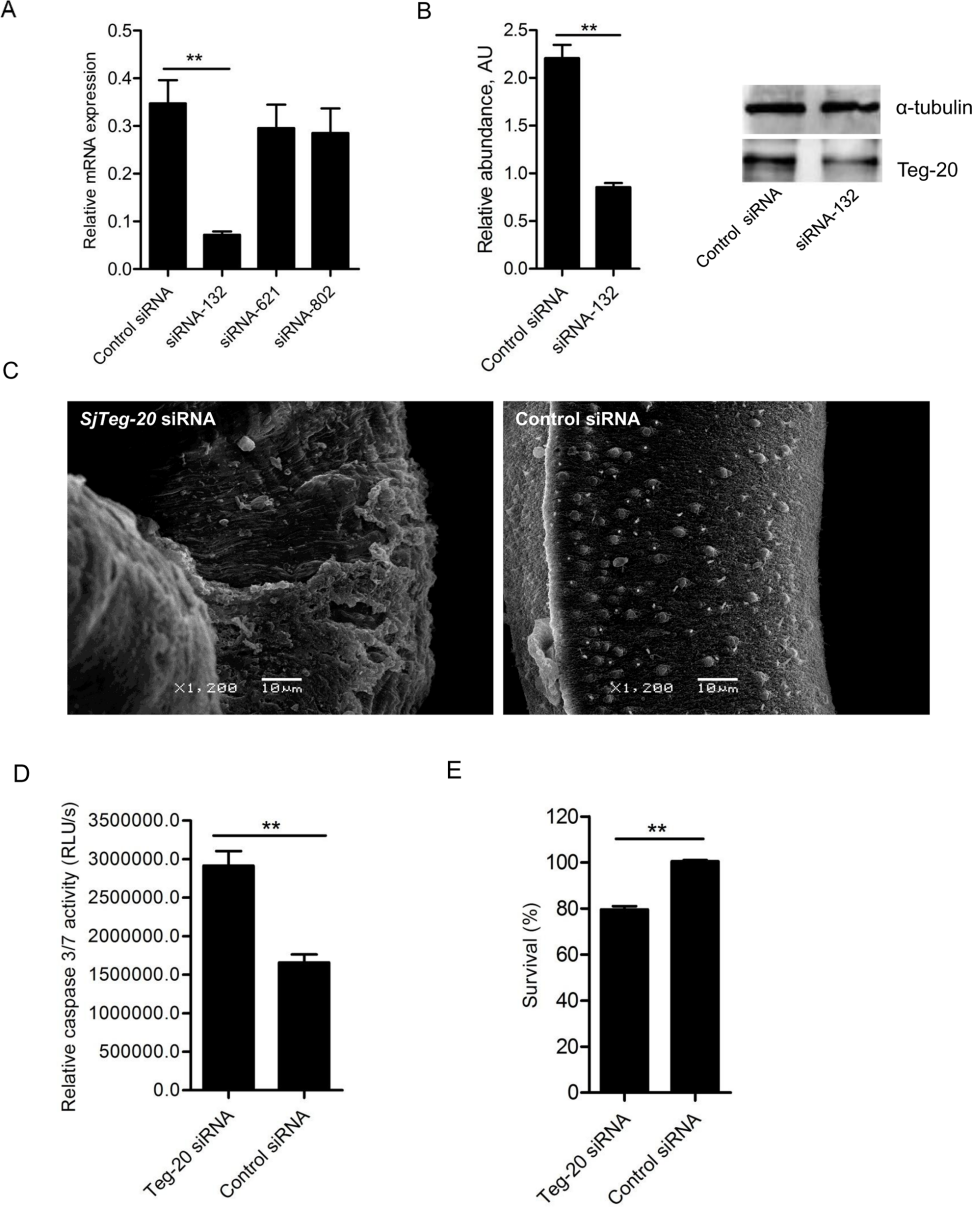
Effects of SjTeg-20 silencing on caspase activity, tegument destruction, and worm mortality. (A) Screening of the best siRNA duplex for inhibiting SjTeg-20. Three siRNA duplexes were electroporated into *in vitro* cultured schistosomes, respectively, and *SjTeg-20* mRNA levels were determined relative to *SjNADH* in *SjTeg-20* siRNA and control siRNA-treated worms at 4 days post electroporation by qRT-PCR. Data illustrate representative results and show the mean and standard errors derived from triplicate experiments. ** *P* ≤ 0.01. (B) Validation of the *SjTeg-20* siRNA-132 duplex for silencing SjTeg-20 using western blot analysis. The densitometry results (arbitrary units [AU]) of the Western blot analyzed by Image J were shown as a bar graph. Each bar represents mean±SEM from triplicate experiments. Representative Western blot result was also shown. ** *P* ≤ 0.01. (C) SjTeg-20 silencing resulted in morphological alterations in the tegument of *S*. *japonicum* as determined by SEM. Data are representative results from at least 15 worms investigated in three independent experiments. (D) SjTeg-20 silencing resulted in increased caspase 3/7 activity of cultured worms (28 d) *in vitro* which were electroporated with *SjTeg-20* siRNA-132. (E) SjTeg-20 silencing increased worm mortality based on Hoechst 33258 dye staining and microscopic observations. Data are representative results and shown as the mean ± standard error from triplicate experiments. ** *P* ≤ 0.01 (Student’s t test, *SjTeg-20* siRNA vs control siRNA).

To further reveal the functions of SjTeg-20, we designed three siRNA duplexes based on the cDNA sequence of *SjTeg-20* for RNAi (S1 Table). All three siRNAs were individually electroporated into adult schistosomes *in vitro*, and qRT-PCR analysis was conducted at 4 days post-treatment to screen for those siRNAs showing the best silencing effect. This applied to siRNA-132, which led to a 79.4% reduction of the transcript level of *SjTeg-20* (Fig 6A). Successful silencing was confirmed at the protein level by western blot analysis (Fig 6B). Next, we used this siRNA duplex to determine the effect of SjTeg-20 silencing at the morphological level. SEM analysis showed that SjTeg-20 silencing led to swelling and detachment of the tegument (Fig 6C). In addition, SjTeg-20 silencing resulted in a significantly increased caspase 3/7 activity (Fig 6D) and in a significant mortality (20%, P = 0.00014) (Fig 6E). Also these results substantiate that SjTeg-20 is an interaction partner of SjIAP.

### SjIAP and SjTeg-20 cooperate to regulate apoptotic processes and maintain the tegument architecture in *S*. *japonicum*

**Fig 7:**
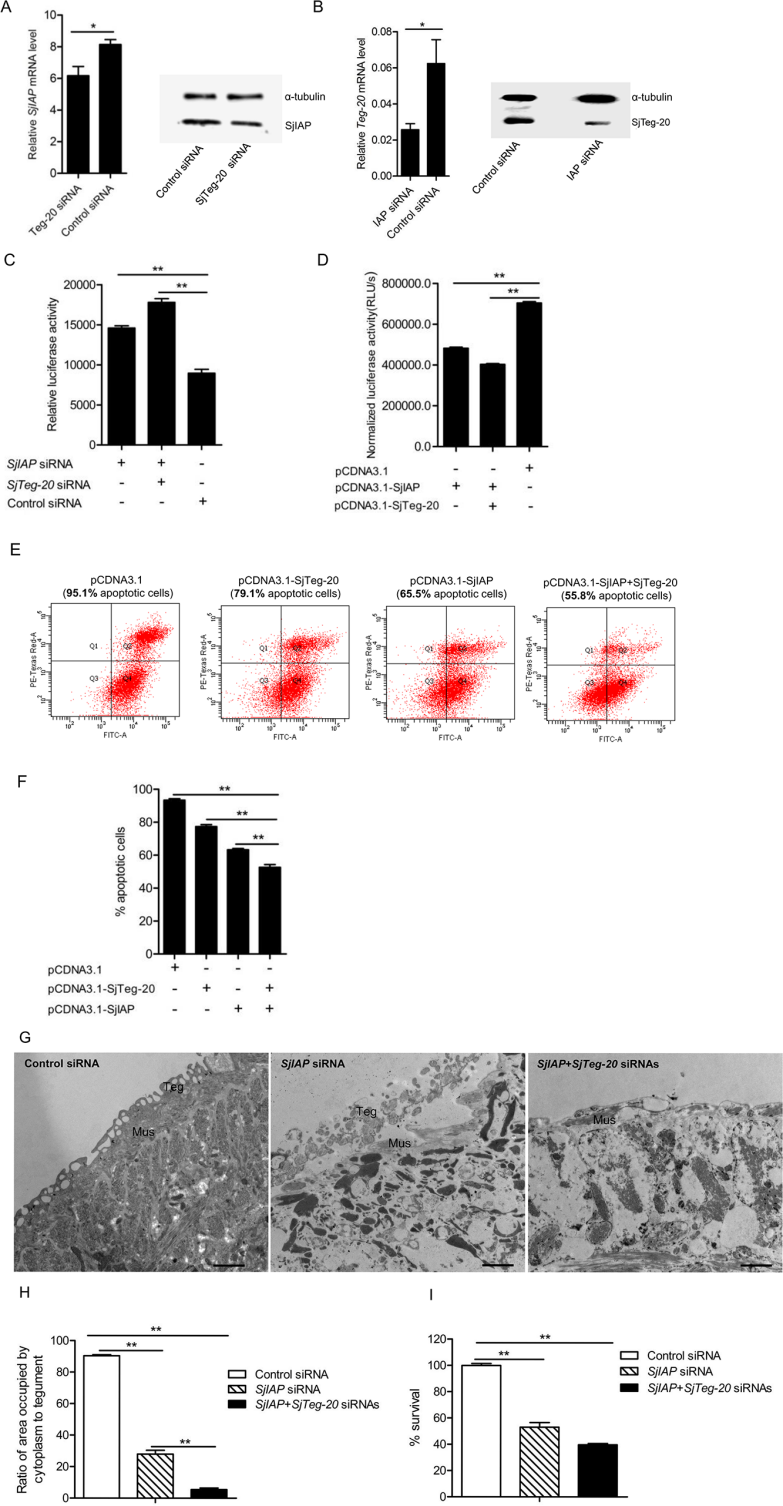
SjIAP and SjTeg-20 synergistically regulate the apoptotic process and maintain the tegument architecture in *S*. *japonicum*. (A) qRT-PCR and Western blot analysis to investigate the effect of *SjTeg-20* silencing on the expression of SjIAP. qRT-PCR data are representative results and shown as the mean ± standard error from triplicate experiments. * *P* ≤ 0.05. (B) qRT-PCR and Western blot analysis of the effect of SjIAP silencing on the expression of SjTeg-20. qRT-PCR data are representative results and shown as the mean ± standard error from triplicate experiments. * *P* ≤ 0.05. (C) Co-silencing of SjIAP and SjTeg-20 significantly elevated caspase 3/7 activity determined from worm protein lysates. Data are representative results and shown as the mean ± standard error from triplicate experiments. ** *P* ≤ 0.01. (D) Effect of co-transfection of recombinant plasmids for expressing SjIAP and SjTeg-20 on the caspase activity in mammalian cells. HEK293T cells were treated with Cyclosporin A for 12 h and then transfected with recombinant plasmids expressing SjIAP and SjTeg-20 (2 μg). At 12 h post transfection, cells were collected for determining caspase 3/7 activity. Data are representative results and show the mean ± standard error from triplicate experiments. ** *P* ≤ 0.01. (E) Effect of co-transfection of the recombinant plasmids expressing SjIAP and SjTeg-20 on cell apoptosis in mammalian cells. HEK293T cells were treated with Cyclosporin A for 12 h, transfected with recombinant plasmids expressing SjIAP and SjTeg-20 (2 μg), stained with Annexin V-FITC kit, and subjected to flow cytometry. Data are representative results from triplicate experiments. (F) Quantitation of apoptosis cells transfected with recombinant plasmids expressing SjIAP and SjTeg-20 in HEK293T cells using flow cytometry. Data are representative result and shown the mean ± standard error from triplicate experiments. ** *P* ≤ 0.01. (G) TEM of morphological alterations in the tegument of *S*. *japonicum* treated with *SjIAP* siRNA and/or *SjTeg-20* siRNA. Data are representative results from at least 15 worms investigated in three independent experiments. Scale bars: 2 μM. (H) Quantification of tegument changes due to co-silencing SjIAP and SjTeg-20 shown in (G). The tegument defects were analyzed using Image J based on the ratio of the area occupied by the tegument cytoplasm to the total area of the tegument. Data show the mean and standard errors derived from four randomly selected parasites. ** *P* ≤ 0.01. Abbreviations: Mus-muscles; teg-surface layer of the tegument. (I) Co-silencing SjIAP and SjTeg-20 resulted in significantly increased worm mortality. At 4 days of post treatment, worms were stained with Hoechst 33258 dye, and the mortality was determined by microscopy. Data are representative results and shown as the mean ± standard error from triplicate experiments. ** *P* ≤ 0.01.

From the data obtained so far we inferred that SjIAP and SjTeg-20 may cooperate to maintain tegument integrity in *S*. *japonicum* by negatively regulating apoptotic processes. To confirm this synergistic effect, we first determined whether the expression of either one of these two molecules is altered when the other has been silenced *in vivo*. As shown in Fig 7A, inhibition of *SjTeg-20* by siRNA resulted in the reduction of the level of SjIAP. Similarly, silencing of *SjIAP* led to a decreased level of SjTeg-20 (Fig 7B). We next investigated synergistic effects on caspase activity when SjIAP and SjTeg-20 were co-silenced. As shown in Fig 7C, SjIAP silencing led to 1.6-fold increase in caspase activity compared to a control, whereas the combination with SjTeg-20 inhibition resulted in a 1.98-fold increase in caspase activity *in vivo*. To further confirm their synergistic effects on apoptotic inhibition, we cloned the *SjIAP* and *SjTeg-20* coding sequences into the eukaryotic expression vector pcDNA3.1. The resulting recombinant plasmids were then transfected into HEK293T cells treated with Cyclosporin A for artificially inducing cellular apoptosis. With this approach we intended to determine whether SjIAP and SjTeg-20 cooperatively inhibit apoptotic process in a heterologous system. The caspase activity assay indicated that transfection of the pCDNA3.1-SjIAP plasmid alone resulted in a 32% reduction of caspase activity compared to that of cells transfected with control plasmids. Moreover, co-transfection of pCDNA3.1-SjIAP and pCDNA3.1-SjTeg-20 further decreased the caspase activity up to 43% compared to transfection with control plasmids (Fig 7D). These results were further corroborated by flow cytometry to detect the proportion of apoptotic cells using Annexin V-FITC staining (Fig 7E and 7F).

Next, we investigated the synergistic effect of co-silencing SjIAP and SjTeg-20 on morphological changes of the tegument in *S*. *japonicum* by TEM analysis. Similar morphological alterations were observed as detected when applying *SjIAP* siRNA alone, including the formation of enlarged vacuoles and appearance of the damaged apical and syncytial cytoplasm in the tegument (Fig 7G). When SjIAP and SjTeg-20 were co-silenced, the external syncytial layer was almost completely removed, and the basal lamina was exposed in the tegument (Fig 7G). In addition, we noted extensive and enlarged vacuoles throughout the surface layer and musculature in the treated worms (Fig 7G). In contrast, no morphological alterations were observed in the worms treated with control siRNAs. The experiment was repeated three times with similar results and representative images are shown (Fig 7G). Further examination of the ratio of the areas occupied by tegument cytoplasm to the total area of the tegument demonstrated that co-silencing SjIAP and SjTeg-20 led to a significant reduction of the area occupied by the tegument cytoplasm (Fig 7H). In addition, we observed that co-silencing SjIAP and SjTeg-20 also significantly increased worm mortality (Fig 7I).

### Silencing SjIAP and SjTeg-20 decreased worm burden and egg production in mice infected with *S*. *japonicum*

**Fig 8:**
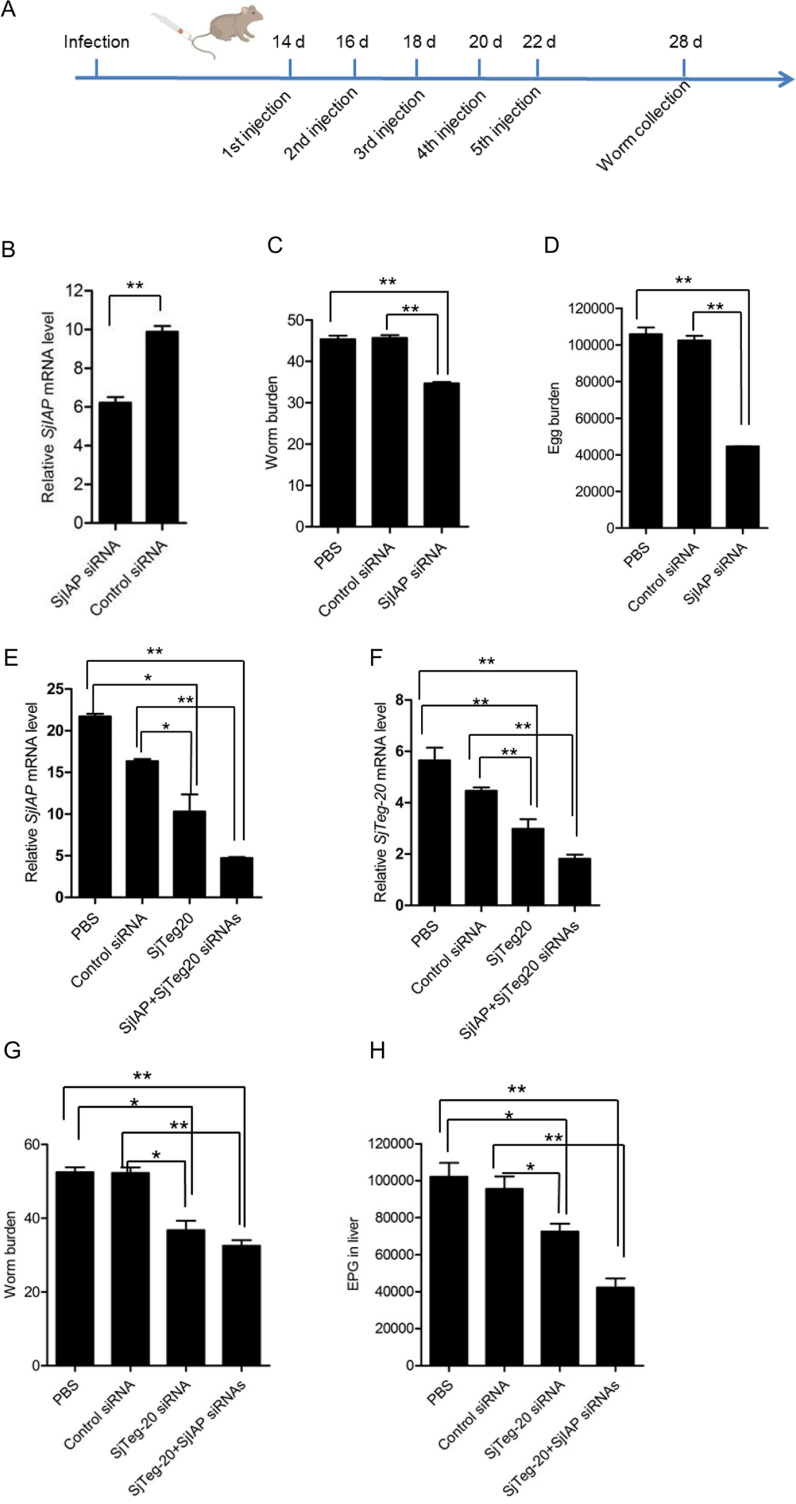
Silencing of SjIAP and SjTeg-20 decreased worm burden and egg deposition in mice *in vivo*. (A) Schematic showing the schedule for siRNA injection in mice. Each treatment included 6 mice (n = 6) (B) qRT-PCR analysis of *SjIAP* mRNA from surviving schistosomes isolated from mice injected with *SjIAP* siRNA by hydrodynamic tail vein injection, determined relative to *SjNADH*. Data are shown as the mean ± standard error and are representative results from triplicate experiments. ** *P* ≤ 0.01. (C) Effect of *SjIAP* siRNA injection on the worm burden in mice. At 14 days post infection, mice were administrated with *SjIAP* siRNA five times with one day interval. At 28 days of post infection, worms were perfused from mice, and the number of worms was counted. ** *P* ≤ 0.01. (D) Effect of *SjIAP* siRNA injection on egg deposition in the liver of mice. ** *P* ≤ 0.01. (E) qRT-PCR analysis of *SjIAP* mRNA levels from schistosomes isolated from mice injected with *SjIAP*/*SjTeg-20* siRNA by hydrodynamic tail vein injection in the biological replicates. Levels of *SjIAP* mRNA relative to *SjNADH* were analyzed by qRT-PCR in surviving worms used for RNA isolation. Data are shown as the mean ± standard error and representative results from triplicate experiments. * *P* ≤ 0.05 and ** *P* ≤ 0.01. (F) qRT-PCR analysis of *SjTeg-20* mRNA level from schistosomes isolated from mice injected with *SjIAP*/*SjTeg-20* siRNAs by hydrodynamic tail vein injection in the biological replicates. Levels of *SjTeg-20* mRNA relative to *SjNADH* were analyzed by qRT-PCR in surviving worms used for RNA isolation. Data are shown as mean ± standard error and representative results from triplicate experiments. ** *P* ≤ 0.01. (G) Effect of *SjIAP*/*SjTeg-20* siRNA injection on the worm burden in mice. At 14 days post infection, mice were administered with *SjIAP*/*SjTeg-20* siRNA as indicated above. At 28 days of post infection, worms were perfused from mice, and the number of worms was counted. * *P* ≤ 0.05 and ** *P* ≤ 0.01. (H) Effect of *SjIAP*/*SjTeg-20* siRNA injection on egg deposition in the liver of mice. * *P* ≤ 0.05 and ** *P* ≤ 0.01.

Given that inhibition of SjIAP or SjTeg-20 affects the survival of *in vitro* cultured schistosomes, we further investigated whether silencing of SjIAP and SjTeg-20 would reduce the worm burden and egg production in a rodent model for *S*. *japonicum* infection. Hydrodynamic tail-vein injection has been shown before by others and us to be a suitable way of delivering siRNAs into mice [31, 32]. Therefore, we adopted this technique in the present study to silence SjIAP in mice infected with *S*. *japonicum* cercariae. Upon five injections (Fig 8A), parasites were perfused and collected. RNA was extracted from surviving worms and transcript levels were analyzed by qRT-PCR. The *SjIAP* mRNA level was significantly decreased in worms collected from mice administered with *SjIAP* siRNA as compared to controls (Fig 8B). In addition, significantly fewer parasites were recovered from the mesenteric veins compared to those of the control siRNA group (Fig 8C). Mice injected with *SjIAP* siRNA showed a 25% (p = 0.00012) reduction in the number of parasites recovered in comparison to the control group. Moreover, there was a significant decrease in the number of eggs deposited in the liver up to 57% in mice administrated with *SjIAP* siRNA (p = 0.00007854) (Fig 8D). When *SjTeg-20* and *SjIAP* siRNAs were co-administered, mRNA levels of both genes decreased significantly compared to the control (Fig 8E and 8F). Furthermore, an additive effect of co-administering was also observed for worm burden and egg production, both further decreased upon applying *SjTeg-20* and *SjIAP* siRNAs compared to the treatment *SjTeg-20* siRNA alone (Fig 8G and 8H).

## Discussion

In many organisms apoptosis is an evolutionarily conserved process for programmed cell elimination. In general, there are two major pathways to apoptosis, the intrinsic and extrinsic pathways. Initially, genomic studies indicated that several molecules involved in apoptosis regulation exist in *S*. *japonicum* [14], and studies applying caspase activity [11, 23], TUNEL staining, DNA fragmentation (a hallmark of apoptosis) [23], and Annexin V staining [5] further supported a role of apoptosis in schistosomes. A recent study characterized the major components of apoptosis pathways in schistosomes and indicated that a Bcl-2 family-regulated mitochondrial cell death pathway exists in schistosomes that is evolutionarily more closely related to that of humans than to *C*. *elegans* [15, 33].

Cell survival requires the active inhibition of apoptosis, which is accomplished by inhibiting the expression of pro-apoptotic factors as well as promoting the expression of anti-apoptotic factors. In mammalians, these inhibitors, including the Bcl-2 family, cytokine response modifier A, and IAPs, were frequently observed in cancer and other diseases to negatively regulate apoptosis. In insects, the control of the apoptosis is dominated by IAPs that function by inhibiting caspases as well as a prosurvival protein (Buffy) and a Bax/Bak ortholog (Debcl/dBok) [34]. The *Drosophila melanogaster* genome contains at least four members of the IAP family: *Drosophila* IAP1 (DIAP1), *Drosophila* IAP2 (DIAP2), DETERIN and *Drosophila* BIR repeat-containing ubiquitin-conjugating (dBRUCE) [16]. Among them, DIAP1 was shown to play essential roles in cell survival by neutralizing apoptosis [35, 36]. Loss-of-function mutations in DIAP1 (thread) led to early embryonic death with a massive caspase-dependent apoptosis [36, 37]. The human genome encodes eight members of the IAP family, which have diverse functions, including regulation of the innate immune response, cell division, and cell death pathways [38]. Among the human IAPs, X-linked IAP (XIAP) and cellular IAP1 and 2 (cIAP1/2) have similar properties with DIAP1, including the capacity to inhibit apoptosis. In *Schistosoma*, beside the Bcl-2 protein [15], we cloned three IAP related proteins (IAP[23], BIRP[25], CIAP[24]) in *S*. *japonicum*. Bioinformatics analysis indicated that two (SjIAP and SjBIRP) of these contain BIR domains, and loss of the CARD as well as RING domains. This is a typical feature of IAPs in humans and mice, suggesting that schistosome IAP proteins may have specific functions [23–25].

Here, we demonstrated that the inhibition of SjIAP significantly increased caspase activity and the number of TUNEL^+^ cells in the tegument of schistosomes. More importantly, silencing SjIAP by siRNA resulted in alterations of the tegument of schistosomes, which was associated with high mortality. To gain more molecular insight into SjIAP-mediated regulation processes, we used co-immunoprecipitation and identified SjTeg-20 as a potential interaction partner. Besides confirming interaction using Y2H, SjIAP and SjTeg-20 were found to exhibit similar transcript profiles at different life stages of *S*. *japonicum*, and they co-localized in the tegument of adult worms. Moreover, silencing one of these two molecules was associated with altered expression of the other gene in *S*. *japonicum*. These results clearly demonstrated that SjIAP may interact and cooperate with SjTeg-20.

In addition, we found that SjTeg-20 suppression led to elevated caspase activity in *S*. *japonicum*, and transfection of recombinant plasmids for expressing SjTeg-20 significantly inhibited cellular apoptosis in a heterologous system. Bioinformatics analysis indicated that SjTeg-20 is a Ca^2+^-binding protein belonging to the EF-hand superfamily. In mammals, the Ca^2+^-binding protein ALG-2 has been documented to regulate apoptosis [39]. Consequently, we have revealed a yet undiscovered function of SjTeg-20 in inhibiting apoptosis in *S*. *japonicum*. In addition, we found that SjTeg-20 was primarily localized in the tegument of *S*. *japonicum*, and its silencing led to morphological defects of the worm tegument. Similarly, in *S*. *mansoni*, SmTeg-20.8 protein, the homolog of SjTeg-20, was shown to localize to the tegument of adult worms [40]. The IgG fraction specific to SmTeg-20.8 killed up to 34% of the schistosomules *in vitro*, and the vaccination of mice with plasmids encoding SmTeg-20.8 resulted in a 30% reduction of the worm burden compared to controls [40]. This suggested that SmTeg-20.8 may have potential for development of a vaccine and represents a candidate drug target against schistosomiasis [40]. Our results support this view by showing that SjTeg-20 silencing resulted in significant worm mortality, reduced worm burden, and decreased egg production in mice infected with *S*. *japonicum*.

Furthermore, we found that co-silencing of SjIAP and SjTeg-20 increased caspase activity in *S*. *japonicum* (Fig 7C) suggesting that cell apoptosis was elevated. Co-transfection of plasmids encoding SjIAP and SjTeg-20 significantly inhibited apoptosis in mammalian cells (Fig 7E and 7F). In addition, we found inhibition of SjIAP/SjTeg-20 also led to the decreased its interacting partner (Fig 7A and 7B). However, it should keep in mind that other proteins and their binding partners may have different situation. These results strongly suggest that SjIAP and SjTeg-20 interaction coordinates processes negatively inhibiting apoptosis in *S*. *japonicum*. Moreover, co-silencing of SjIAP and SjTeg-20 synergistically increased the rate of parasite mortality *in vitro* while devastating the tegument architecture, further supporting the assumption that the SjIAP/SjTeg-20 tandem may play critical roles for maintaining tegument integrity.

Besides supporting role for nutrient uptake, the schistosome tegument is considered to play essential roles in supporting survival in the hostile environment within the bloodstream of the final host [41–43]. We previously reported that immunization with SjIAP, incorporated by adenovirus transfer, conferred moderate protection against *S*. *japonicum* infection in mice, resulting in an average of 37.95% worm reduction and 31.7% egg reduction [44]. Given that vaccination of mice with plasmids encoding SmTeg-20.8 resulted in a 30% reduction of the worm burden in mice infected with *S*. *mansoni* [40], it is worth exploring whether co-vaccination of SjIAP and SjTeg-20 or the combined expression of SjIAP and SjTeg-20 could lead to an additive effect.

Taken together, our study clearly demonstrates the involvement of SjIAP in maintaining tegument integrity by inhibiting cellular apoptosis in *S*. *japonicum*. These findings highlight a novel function of SjIAP in flatworms, besides caspase activity inhibition it warrants the maintenance of tegument integrity in *S*. *japonicum*. Moreover, SjTeg-20 was identified as an interaction partner of SjIAP also playing a role in apoptosis regulation in *S*. *japonicum*. Therefore, a complex of SjIAP and SjTeg-20 may safeguard the integrity of the tegument by negatively regulating cellular apoptosis. Our findings provide new insight into the role of SjIAP to support schistosome survival in the final host. Importantly, since paired adult schistosomes can parasitize in a final host for several decades with thousands of eggs produced daily, which triggers the primary response of human infection and pathological reactions, our study contributes to approaches finding novel strategies against human schistosomiasis.

## Materials and methods

### Ethics statement

All animal experiments were carried out strictly according to the recommendations in the Guide for the Care and Use of Laboratory Animals of the Ministry of Science and Technology of the People’s Republic of China. All animal procedures were approved by the Institutional Animal Care and Use Committee (IACUC) of the Shanghai Veterinary Research Institute, Chinese Academy of Agricultural Sciences and by the Animal Management Committee and the Animal Care and Use Committee of Shanghai Science and Technology Commission of Shanghai municipal government for the Shanghai Veterinary Research Institute, Chinese Academy of Agricultural Sciences, China (Permit number:SYXK 2016-0010).

### Animals and parasites

The life cycle of *S*. *japonicum* (Anhui isolate) was maintained in New Zealand rabbits (Shanghai Ling Chang Biological Technology Co., Ltd, Shanghai, China) and BALB/c mice (Shanghai SLAC Laboratory Animal Co.,Ltd, Shanghai, China) along with *Oncomelania hupensis* (Center of National Institute of Parasitic Disease, Chinese Center for Disease Control and Prevention, Shanghai, China) as the snail host. Unless indicated otherwise, New Zealand rabbits and BALB/c mice were infected with approximately 1,000 and 100 cercariae, respectively, by inoculating the shaved abdominal skin surface with a moist cercarial paste. To collect different developmental stages of schistosomes, the parasites were perfused from infected rabbits at 7, 14, 21, 28, and 35 days post-infection, respectively. Eggs were isolated from the rabbit livers by gentle homogenization of infected livers in 1.5% saline followed by sequential filtering through screens as described previously [45]. The cercariae of *S*. *japonicum* were collected from infected *Oncomelania hupensis* snails (Center of National Institute of Parasitic Disease, Chinese Center for Disease Control and Prevention, Shanghai, China) placed in tubes filled with distilled water. After the snails were exposed to bright light for 12 h, the cercariae of *S*. *japonicum*, which normally stick to the water surface, were collected by shaking off the top water into a beaker with medium. The parasites were snap-frozen and stored in liquid nitrogen until use.

### RNA isolation and SjIAP expression analysis

Total RNA was extracted from *S*. *japonicum* samples using TRIzol reagent (Invitrogen) according to the manufacturer’s protocol. The isolated RNA was quantified using a Nanodrop ND-1000 spectrophotometer (Nanodrop Technologies, Wilmington, DE). The levels of *SjIAP* mRNA at different stages were analyzed using qRT-PCR by using primers specific to *SjIAP* (forward primer: 5'CGGATCAACCTGAAGCGTGT3', reverse primer: 5'ATTACCCCAGGAAGACCACG 3') and primers for the *S*. *japonicum* nicotinamide adenine dinucleotide (*NADH*) dehydrogenase gene (forward primer: 5'CGAGGACCTAACAGCAGAGG 3'; reverse primer: 5'TCCGAACGAACTTTGAATCC 3') were used as an internal control for normalization. qPCR was performed using the SYBR Premix Ex Taq (TaKaRa, China). A 15-μl reaction mixture contained 1 μl of cDNA, 7.5 μl of 2 × SYBR Primer Ex TagII (TaKaRa, China), 6 μl of H_2_O, and 0.5 μl primers (10 μM). The reactions were amplified in a Master cycler ep realplex (Eppendorf, Germany) real-time PCR detection system, using the following thermal cycling profile: 95 °C for 5 min, followed by 40 cycles of amplification (94 °C for 5 s, 62°C for 30 s, 72°C for 20 s). Relative mRNA expression was calculated using the 2^−ΔCt^ method [46].

### Schistosome culture and electroporation

Adult *S*. *japonicum* were collected from mice as indicated day, and cultured in a 12-well flat bottom plate containing 2 mL complete RPMI-1640 medium containing 2 g/L glucose, 0.3 g/L l-glutamine, 2.0 g/L NaHCO_3_, 15% fetal bovine serum (Gibco, USA), and 5% penicillin/streptomycin (10,000 units penicillin and 10 mg/streptomycin in 0.9% NaCl) (Gibco) in a humidified 5% CO_2_ chamber at 37°C. The cultured schistosomes were then electroporated with *SjIAP* siRNA and/or *SjTeg-20* siRNA and control siRNA (3 μg per experiment, Shanghai GenePharma, China) (S1 Table) at 125 V, 20 ms, 1 pulse in 200 μL RPMI 1640 medium. Schistosomes were then transferred into a 12-well cell culture plates containing 2 mL fresh medium. The parasites were usually collected at 4 days post electroporation for qRT-PCR analysis, western blot, caspase activity assays, and electron microscopy as described below unless otherwise indicated.

### qRT-PCR analysis to determine transcript levels of *SjIAP* and several caspases in SjIAP silenced parasites

Total RNA was extracted from treated and control parasites using TRIzol reagent (Invitrogen), and the isolated RNA was further quantified as described above. The levels of *SjIAP*, *caspase 2*, *caspase 3*, or *caspase 7* mRNA were analyzed using qRT-PCR by using the specific primers (*SjIAP*: forward primer: 5'CGGATCAACCTGAAGCGTGT3', reverse primer: 5'ATTACCCCAGGAAGACCACG 3'; *Sjcaspase2*: forward primer: 5'TGCTAGCTGGGAAACCCAAG3', reverse primer: 5'TTCACGAGAATTCGACGGCA 3'; *Sjcaspase3*: forward primer: 5'GGTTGCACGTGTAGTAGCCT3', reverse primer: 5'TCTCCCGCGTTTATCACCTG 3'; *Sjcaspase7*: forward primer: 5'GTCCGTTTCCTGTGCTTCCT 3', reverse primer: 5'CACAAGCGAAGCAGTCATGG 3') and primers for the *S*. *japonicum* nicotinamide adenine dinucleotide (*NADH*) dehydrogenase gene were used as an internal control for normalization. qPCR was carried out using the SYBR Premix Ex Taq (TaKaRa, China). A 15-μl reaction mixture contained 1 μl of cDNA, 7.5 μl of 2 × SYBR Primer Ex TagII (TaKaRa, China), 6 μl of H_2_O, and 0.5 μl primers (10 μM). The reactions were amplified in a Master cycler ep realplex (Eppendorf, Germany) real-time PCR detection system, using the following thermal cycling profile: 95 °C for 5 min, followed by 40 cycles of amplification (94 °C for 5 s, 62°C for 30 s, 72°C for 20 s). Relative mRNA expression was calculated using the 2^−ΔCt^ method [46]

### Western blot analyses

Lysates were prepared from parasites treated with *SjIAP/SjTeg-20* siRNA or control siRNA, and the proteins were quantified by Bradford method. Equal amounts of protein lysates from siRNA-treated worm groups and control groups were subjected to sodium dodecyl sulfate polyacrylamide gel electrophoresis (SDS-PAGE; 10% resolving gel) and then electrotransferred onto polyvinylidene difluoride membranes (BioRad, USA). Non-specific protein–protein interactions were blocked using 5% non-fat dry milk (Sangon Biotech, China) in phosphate buffered saline (PBS; pH = 7.4) containing 0.1% Tween 20 (PBST; Sigma-Aldrich). The membrane was incubated for 1 h at room temperature with primary antibodies/sera against SjIAP and SjTeg-20 (1:2000 dilutions)(Shanghai Veterinary Research Institute, China) and anti-alpha tubulin (CoWin Biosciences, China) diluted 1:2000 in blocking buffer, and washed five times for 5 min each in 0.1% PBST. The membrane was then incubated with the secondary antibody horseradish peroxidase-conjugated goat anti-rabbit/mouse immunoglobulin (Ig)G (CoWin Biosciences, China), diluted to 1:5000 in PBS, for 1 h. The membrane was developed using Immobilon Western Kit according to the manufacturer's instructions (Millipore).

### Co-immunoprecipitation experiments

Adult schistosomes (~200 mg, 24 days post-infection) were lysed in 600 μL lysis buffer containing 25 mM Tris (pH 7.4), 150 mM KCl, 0.5% NP-40, 2 mM ethylenediaminetetraacetic acid (EDTA), 0.5 M dithiothreitol, and protease inhibitors, and then centrifuged at 5,000 ×*g* for 10 min at 4°C. The pull-down assay was carried out using a Dynabeads Protein G Immunoprecipitation Kit (Invitrogen) according to the manufacturer’s instructions with our previously developed polyclonal antibodies for SjIAP [23]. In brief, approximately 200 μL of anti-SjIAP serum and pre-immune serum (Shanghai Veterinary Research Institute, China) were coupled with 3.0 mg Dynabeads, respectively. Then, 400 μL of the worm lysate was incubated with the antiserum-treated beads for 15 min at room temperature, and the Dynabeads were washed three times in 400 μL of the wash buffer provided with the kit. Finally, the pull down product was recovered with elution buffer, and the eluted solutions were analyzed by SDS-PAGE followed by sliver staining as described in our previous study [47]. The different bands as compared to those obtained through pull down by control serum were excised for in-gel digestion of the proteins.

### In-gel digestion

Proteins were in-gel digested with trypsin overnight, and the peptides were extracted according to the method of Shevchenko et al. [48] with a few minor modifications. In brief, the peptides were extracted and then were concentrated in a Speed-Vac to 10 μL, cleaned, and further desalted with C18 Zip-Tips (Millipore). The desalted peptides were dried completely in a Speed-Vac and re-dissolved in 0.6 μL of 0.1% trifluoroacetic acid for liquid chromatography tandem mass spectrometry (LC-MS/MS) analysis.

### LC–MS/MS analysis

The digested peptides were subjected to LC-MS/MS analysis using an LTQ linear IT mass spectrometer (Thermo, San Jose, CA, USA). The system was fitted with a C18 RP column (180 μm × 150 mm, BioBasic C18.5 μm; Thermo Hypersil-Keystone, USA). Mobile phase A was 0.1% formic acid in water and mobile phase B was 0.1% formic acid in acetonitrile). The tryptic peptide mixtures were eluted using a gradient of 2–98% B over 120 min. The LTQ linear IT mass spectrometer was set so that one complete MS scan was followed by three MS/MS scans on the most intense ions from the MS spectrum with the following dynamic exclusion settings: repeat count, 2; repeat duration, 30 s; exclusion duration, 90 s.

### Protein identification

The acquired MS/MS spectra were searched against the protein database for schistosome proteins using the Turbo SEQUEST program in the BioWorks 3.0 software suite. An accepted SEQUEST result was defined as having a DCn score of at least 0.1 and a cutoff of Rsp 4. A singly charged peptide was required to be tryptic; the cross-correlation score (X corr) at least 1.9. Tryptic or partially tryptic peptides with a charge state of 12 were required to have an Xcorr value of at least 2.2. Triply charged tryptic or partially tryptic peptides with a 13 charge state was accepted if the Xcorr value was 3.75 [49].

### Immunohistochemistry

Immunohistochemistry was performed to determine the distribution of SjTeg-20 and SjIAP in adult worms of *S*. *japonicum* in accordance with the standard protocol. In brief, *S*. *japonicum* adult worms (28 days) were fixed in 4% formaldehyde solution at 4°C overnight. Paraffin sections were prepared, which were then deparaffinized with xylene and rehydrated through different concentrations of ethanol immersion. Endogenous peroxidase activity was quenched with 0.3% (v/v) hydrogen peroxide in methanol for 15–20 min, followed by three 5-min washes with PBS. The sections were then blocked with 10% (v/v) rabbit serum (Sigma-Aldrich) in PBS for 1 h, followed by overnight incubation at 4°C with the primary antibodies /sera against SjIAP and SjTeg20 (1:200 dilutions) in PBS containing 3% (w/v) BSA. The negative control was established by replacing the primary antibody with pre-immune mice/rabbit serum (Shanghai Veterinary Research Institute). After three 5-min washes with 0.02% PBST, the sections were treated with biotinylated goat anti-rabbit/mouse antibody (Abcam) for 20 min at room temperature, followed by three additional 5-min washes with PBST. The sections were then incubated with streptavidin-horseradish peroxidase for 20 min at room temperature, followed by repeated washes as described previously. The reaction product was visualized with 3,3-diaminobenzidine (DAB) at room temperature for 5 min. The sections were counterstained with hematoxylin for 30 s and rinsed with Milli-Q water, immediately dehydrated by sequential immersion in gradient ethanol and xylene, and then mounted with Permount on coverslips. Images were captured under a light microscope equipped with a digital camera.

### Yeast two-hybrid analyses

A yeast two-hybridization assay from the DUAL membrane starter kits (Dualsystems Biotech) was used to validate the interaction between SjIAP and SjTeg-20 in yeast cells. In brief, the *SjTeg-20*-encoding sequence was amplified via PCR using the primer pair forward 5′AAGGCCATTACGGCCATGGAACCATTTGTTCAAGTC3′, reverse 5′CCGGCCGAGGCGGCCCCTTCACCGGTCAATTCTTCGT3′, each containing an *Sfi*I restriction site, and cDNA was reverse-transcribed from total RNAs and used as a template for PCR [50]. The PCR products were cloned into the bait pBT3STE vector (Dualsystems Biotech) via *Sfi*I. In addition, the specific primers for *SjTeg-20* (forward 5′AAGGCCATTACGGCCATGGAACCATTTGTTCAAGTC3′, reverse 5′CCGGCCGAGGCGGCCTTCACCGGTCAATTCTTCGT3′; containing *Sfi*I restriction sites) were used to amplify the cDNA fragment to construct recombinant plasmids for the pPR3N vector. The *SjIAP*-encoding sequence was also amplified by PCR using the primers forward 5'AAGGCCATTACGGCCATGTCTTATTTTCAGAACCTGTCAAAT3', reverse 5'CCGGCCGAGGCGGCCTTTTGGAACATTATTGCTGTGAGTT3', containing *Sfi*I restriction sites, for constructing the prey pPR3N-SjIAP plasmid and bait pBT3STE-SjIAP plasmid. The recombinant plasmids including pBT3 STE -SjTeg-20/SjIAP and pPR3N-SjIAP/SjTeg-20 were further confirmed by PCR amplification and sequencing to verify the correct insertions. The plasmids were further analyzed to determine expression of the target genes by activating the reporter genes *ADE2* and *HIS3*.

Different combinations of plasmids (pNubG-Fe65 and pTSU2-APP, positive control; pPR3N and pTSU2-APP, negative control; pBT3 STE and pPR3N, empty plasmids; pBT3 STE -SjTeg-20 and pPR3N-SjIAP, function evaluation; pBT3 STE -SjIAP and pPR3N-SjTeg-20, function evaluation) were transformed into NMY32 yeast cells. The transformed yeast cells were further cultured with different dilutions (20-fold dilutions) onto different plates (SD-Leu-Trp and SD-Leu-Trp-His-Ade+X-Gal).

### SjTeg-20 transcription analysis

Total RNAs was extracted from *S*. *japonicum* different stages (eggs, cercariae, 7 days, 14 days, 21 days, 28 days, 35 days) using TRIzol reagent (Invitrogen) according to the manufacturer's protocol. The obtained RNA was quantified as described above. The levels of SjTeg-20 mRNA at different stages were analyzed using real-time RT-PCR by using primers specific to *SjTeg-20* (forward primer: 5'GTTCAAGTCTTCTTCGCTAT 3', reverse primer: 5'CATTTTGTAACCATCATTTC 3') and primers for the *S*. *japonicum* nicotinamide adenine dinucleotide (*NADH*) dehydrogenase gene were used as an internal control for normalization. Real-time PCR was performed using the SYBR Premix Ex Taq (TaKaRa, China). A 15-μl reaction mixture contained 1 μl of cDNA, 7.5 μl of 2 × SYBR Primer Ex TagII (TaKaRa, China), 6 μl of H_2_O, and 0.5 μl primers (10 μM). The reactions were amplified in a Master cycler ep realplex (Eppendorf, Germany) real-time PCR detection system, using the following thermal cycling profile: 95 °C for 5 min, followed by 40 cycles of amplification (95 °C for 5 s, 60 °C for 30 s, 72 °C for 20 s). Relative mRNA expression was calculated using the 2^−ΔCt^ method [46]

### Cloning, expression, and purification of SjTeg-20 in *Escherichia coli* and generation of antiserum

Cloning, expression, and purification of *SjTeg-20* were performed according to the standard procedures. Briefly, expression plasmids containing full-length open reading frame of *SjTeg-20* were constructed by PCR-based amplification and subsequent insertion of the corresponding fragment into a pET28a(+) expression vector (Novagen, Germany). Restriction recognition sites of *Bam*HI (5′end) and *Hin*dШ (3′end) were introduced by PCR using specific primers (forward primer: 5'GCGGATCCATGGAACCATTTGTTCAAGT3'; reverse primer: 5'GGAAGCTTTCATTCACCGGTCAATTCTT3') with template of cDNA derived from total RNA. Recombinant SjTeg20 was expressed in transformed *E*. *coli* BL21 (DE3) cells carrying pET28a(+)-SjTeg-20 vector with an induction of 1.0 mM IPTG at 37°C. Soluble recombinant SjTeg-20 (rSjTeg-20) was purified by a Ni-NTA His* Band Purification Kit following the manufacturer’s instructions (Novagen, Germany). The eluted protein fractions were analyzed by 15.0% SDS–PAGE gel. For generation of polyclonal antibodies, mice were intramuscularly immunized with 50 μg recombinant SjTeg-20 emulsified with 206 adjuvant (Sigma-Aldrich), followed by two boosts at 3-week interval. Seven days after the last boost, mice were bled, and the sera were collected and stored at −20°C.

### Cell culture, plasmid construction, and transfection

HEK293T cells (Human embryonic kidney 293 cells and were obtained from Center for Type Culture Collection of Chinese Academy of Sciences, Shanghai, China) were cultured at 37°C in RPMI 1640 complete medium (Invitrogen) supplemented with 5% fetal bovine serum (Gibco) and 100 μg/mL penicillin–streptomycin (Gibco) in a humidified atmosphere containing 5% CO_2_. HEK293T cells were seeded at a density of 2.5 × 10^5^ cells per 24-well plate. Recombinant plasmids encoding *SjIAP* and *SjTeg-20* were constructed by cloning the full-length cDNA encoding *SjIAP* and *SjTeg-20* into a pCDNA3.1 vector (Invitrogen) under the cytomegalovirus promoter using the restriction enzymes *Bam*HІ and *Eco*RІ. The primers for *SjIAP* (forward primer 5′TAGGATCCGCGATGTCTTATTTTCAGAACCT3′; reverse primer 5′TAGAATTCCGGTTATTTTGGAACATTATTGCT3′) and *SjTeg-20* (forward primer 5′TAGGATCCGCGATGGAACCATTTGTT3′; reverse primer 5′TAGAATTCCGGTCATTCACCGGTCAATT3′) were used to amplify the fragments and introduce the restriction sites. Each recombinant plasmid was confirmed by sequencing to verify the correct insertion. All recombinant plasmids were prepared using the Tiangen endofree midi plasmid isolation kit (Tiangen, China) according to the manufacturer's instructions. After overnight culture, HEK293T cells were treated with 10 μg/mL Cyclosporin A (Targetmol) for 12 h. The recombinant plasmids and control plasmids (2 μg) were transfected into HEK293T cells after complex formation with Lipofectamine 2000 (Invitrogen), respectively. In brief, Lipofectamine 2000 and the plasmids were mixed together in OptiMem I reduced serum medium (Invitrogen), and complex formation was allowed to proceed for 30 min at room temperature, followed by transfection into HEK293T cells to determine caspase 3/7 activity as described below.

### Caspase 3/7 activity

Worms or HEK293T cells were lysed with lysis buffer containing an EDTA-free inhibitor (Roche, Switzerland). Caspase activity in the lysates was measured using a Glo 3/7 Assay kit (Promega, Madison, WI, USA) and a luminometer (Berthold, Germany). A negative control included 100 μL of lysis buffer alone. The protein concentration of each reaction solution was assessed using a Beyotime BCA protein assay kit (Shanghai, China).

### Flow cytometry analysis

At 12 h post-transfection, the cells were detached from the culture plates with 0.5% trypsin treatment. The cells were then spun down by centrifugation and further washed with PBS three times. The cells were stained with Annexin V-FITC Apoptosis Detection Kit according to the product manual (Beyotime Biotechnology, China). In brief, 1 × 10^5^ cells were incubated with 195 μL Annexin V-FITC binding buffer, and then 5 μL Annexin V-FITC and 10 μL propidium iodide staining buffer were added. After incubation at 20–25°C for 15 min in the dark, the cells were analyzed using a BD FACSAria II system (BD Biosciences, Mountain View, CA, USA). Each measurement contained a defined number of 1× 10^4^ cells. Data were analyzed using FACSDiva software (BD Biosciences, Mountain View, CA, USA).

### Viability determination by Hoechst staining

At 4 days post treatment, parasites were stained with 1 μg/ml of Hoechst 33258 dye for 10min and then worm viability was examined under a fluorescence microscopy (Olympus, Tokyo, Japan) as described previously [51]. Worm mobility and survival were observed under an inverted microscope (Olympus, Japan) at the indicated times.

### Electron microscopic analyses

Worms were collected, washed with PBS (pH 7.4) three times, and then fixed with 4% paraformaldehyde and 2.5% glutaraldehyde (System Biosciences, Mountain View, CA, USA) at 4°C for 48 h, followed by osmium tetroxide (System Biosciences, Mountain View, CA, USA) at room temperature. Subsequently, the parasites were dehydrated with increasing concentrations (from 30% to 100%) of acetone (TiTan, Shanghai, China) for incubating. For SEM, the samples were freeze-dried and coated with platinum (System Biosciences) by sputtering with a plasma multicoater (PMC-5000; Meiwafosis, Tokyo, Japan). For TEM, the dehydrated samples were embedded into resins (TiTan, Shanghai, China) before thin sections were cut and transferred to metal grids (Agar Scientific, Essex, UK). Uranyl acetate (System Biosciences) was used for post-section staining. Images were captured with a SEM microscope (JSM-6390LV, JEOL, Tokyo, Japan) in high-vacuum mode with an accelerating voltage of 2–10 kV, or with a TEM microscope (Hitachi H-7600, Tokyo, Japan) with an accelerating voltage of 80 kV.

### Schistosomiasis animal model

Four- to six-week-old male BALB/c mice (mean weight 25 ± 2 g) were purchased from the Shanghai SLAC Laboratory Animal Co.,Ltd and randomly divided into three groups for one experiment: control treatments including PBS and control siRNA and *SjIAP* siRNA treatment and four groups for other experiment: control treatments including PBS and control siRNA, *SjTeg-20* siRNA, *SjTeg-20*+*SjIAP* siRNAs. Each group includes 6 mice and each mouse was challenged with 80 ± 5 normal *S*. *japonicum* cercariae via abdominal skin penetration.

### Hydrodynamic tail vein injection

Starting at 14 days post-infection, each mouse in each group was injected with 0.8 mL of PBS, control siRNA (40 μg/mL), *SjTeg-20/SjIAP* siRNA (40 μg/mL), or the mixture of *SjTeg-20*+*SjIAP* siRNAs (40 μg/mL)via the tail vein, respectively. All of siRNAs were chemically synthesized by Shanghai GenePharma of China. Four additional injections were performed at 16, 18, 20, and 22 days post-infection. At 28 days post-infection, the parasites were perfused and the eggs in liver were counted as described below. The total RNA was isolated from survival worms for qRT-PCR analysis.

### Worm burden and egg counts

At 28 days post-infection, the parasites in each mouse of the four groups were gently perfused using sterile PBS, and the number of single and paired parasites was microscopically counted. Surviving parasites were analyzed for the expression of target genes at the transcript level by qRT-PCR. Egg counts and worm burden were determined as described elsewhere [52].

### Statistical analysis

Differences between groups were assessed for statistical significance using the Student’s t-test (GraphPad Prism Software, CA, USA). A statistically significant difference for a particular comparison was defined as *P* ≤ 0.05.

## Supporting information

S1 Fig MS maps of protein identification. (PDF)

S2 Fig Molecular cloning of SjTeg-20 and recombinant protein purification. (A) Agarose gel analysis of PCR product for amplifying SjTeg-20. (B) SDS-PAGE analysis of the expression of recombinant SjTeg-20 in *E*. *coli*. 1. *E*. *coli* transfected with recombinant plasmid encoding SjTeg-20; 2. *E*. *coli* transfected with control plasmid; 3. Protein marker. (C) SDS-PAGE analysis of purified recombinant SjTeg-20. (D) Western blot analysis of sera against recombinant SjTeg-20. The arrow indicates the target product.
(TIF)

S1 Table List of siRNA duplexes used in the present study. (DOCX)
