# Supplementary material for: Schistosoma japonicum IAP and Teg20 safeguard tegumental integrity by inhibiting cellular apoptosis
Source: PLoS Negl Trop Dis. 2018 Jul 25;12(7):e0006654. doi: 10.1371/journal.pntd.0006654 (PMC6078320; doi:10.1371/journal.pntd.0006654)

DTA: COIP_IAP.3692.3692.1
Precursor ion: 801.56
Mass type: Average
Mod's: (M* +15.9994) C=160.1652

Ion series for charge: +1

AA	A ions	B ions	B* ions	Bo ions	C ions	Y ions	Y* ions	Yo ions	Z ions
D		116.10							
Q		**244.23**				**686.82**			
A		**315.30**				**558.69**			
V		**414.43**				**487.62**			
Q		**542.56**				**388.48**			
L		**655.72**				**260.35**			
K						147.20			

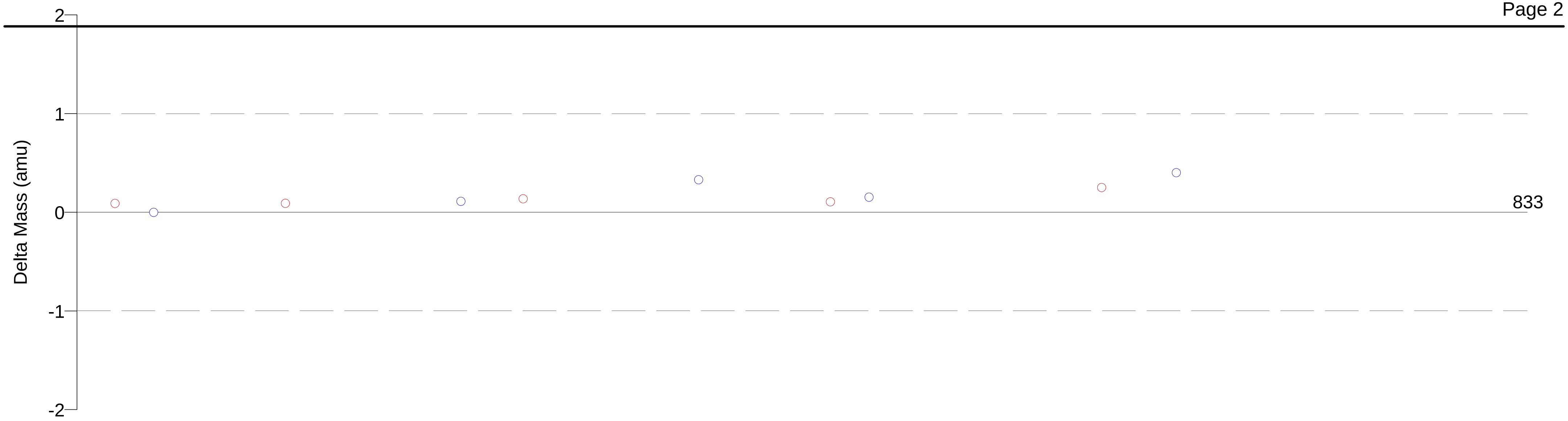

#3692-3692 NL: 6.73E2

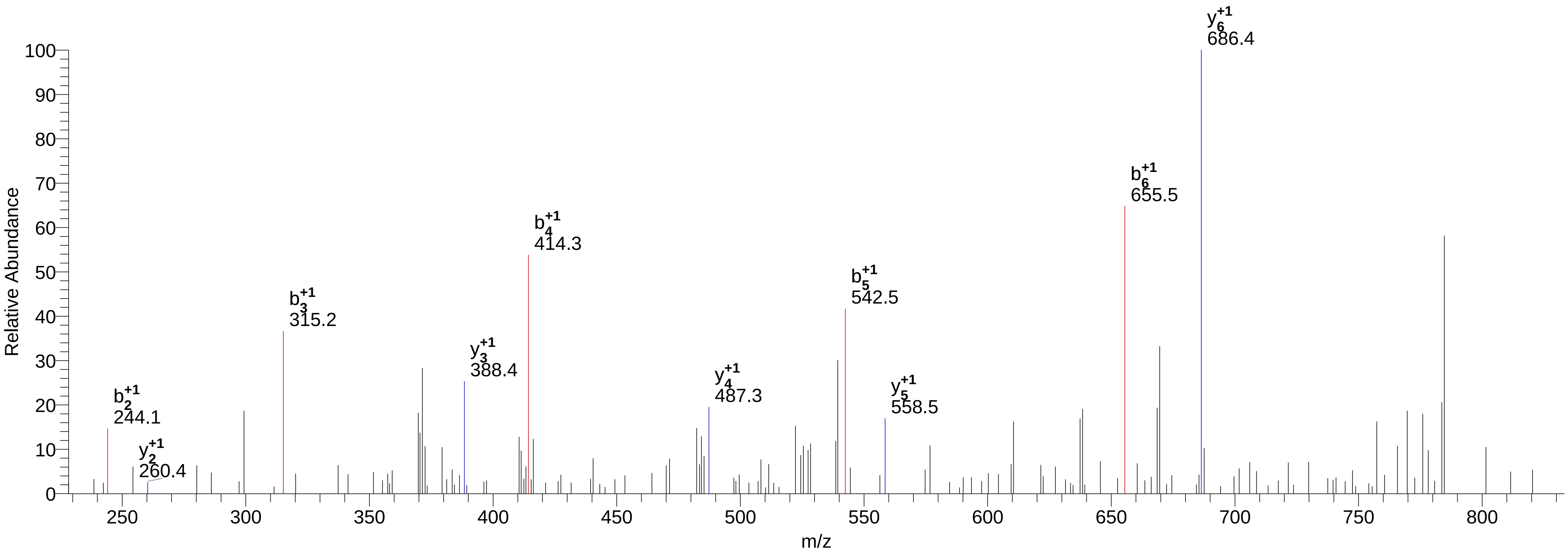

DTA: COIP_IAP.3768.3768.1
Precursor ion: 801.59
Mass type: Average
Mod's: (M* +15.9994) C=160.1652

Ion series for charge: +1

AA	A ions	B ions	B* ions	Bo ions	C ions	Y ions	Y* ions	Yo ions	Z ions
D		116.10							
Q		244.23				686.82			
A		315.30				558.69			
V		414.43				487.62			
Q		542.56				388.48			
L		655.72				260.35			
K						147.20			

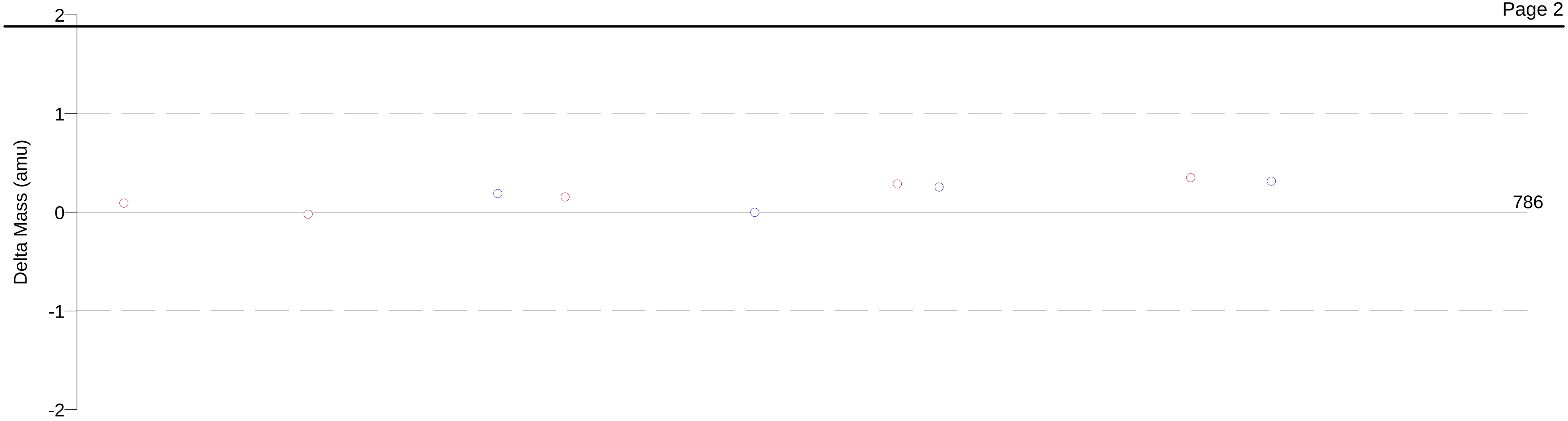

#3768-3768 NL: 7.35E2

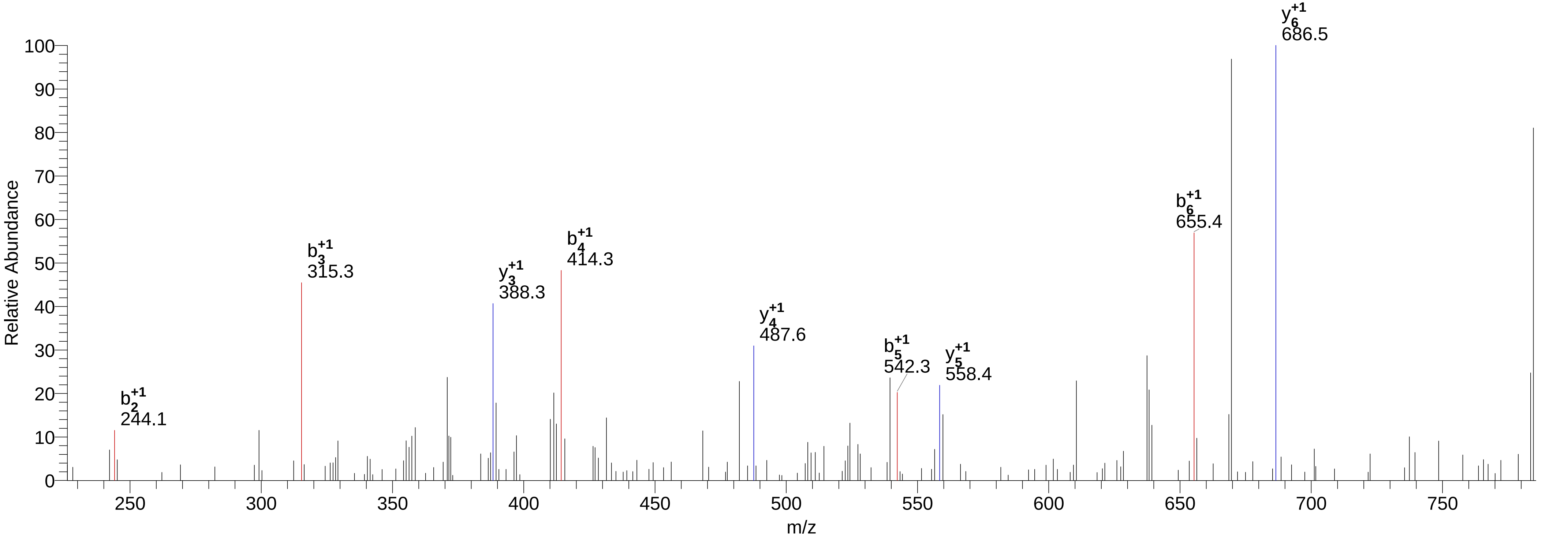

DTA: COIP_IAP.6173.6173.2
Precursor ion: 868.64
Mass type: Average
Mod's: (M* +15.9994) C=160.1652

Ion series for charge: +1

AA	A ions	B ions	B* ions	Bo ions	C ions	Y ions	Y* ions	Yo ions	Z ions
F		148.18							
C		308.35				1590.86			
E		437.46				1430.70			
V		536.59				1301.58			
L		649.75				1202.45			
G		706.80				1089.29			
L		819.96				1032.24			
S		907.04				919.08			
P		1004.15				832.00			
A		1075.23				734.89			
Q		1203.36				663.81			
A		1274.44				535.68			
V		1373.57				464.60			
A		1444.65				365.47			
M*		1591.85				294.39			
K						147.20			

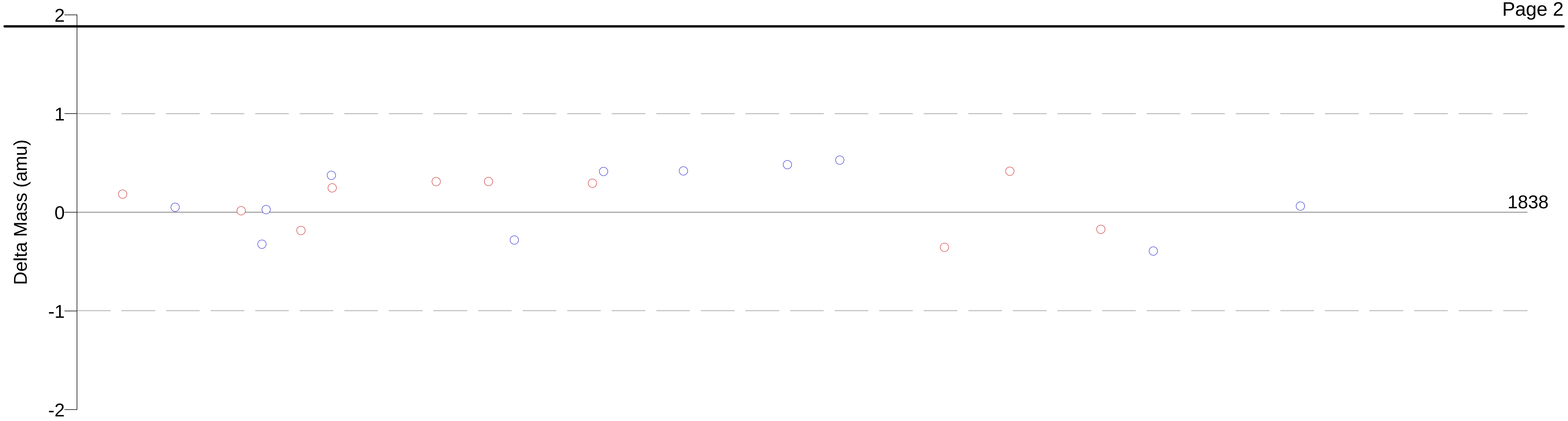

#6173-6173 NL: 1.04E3

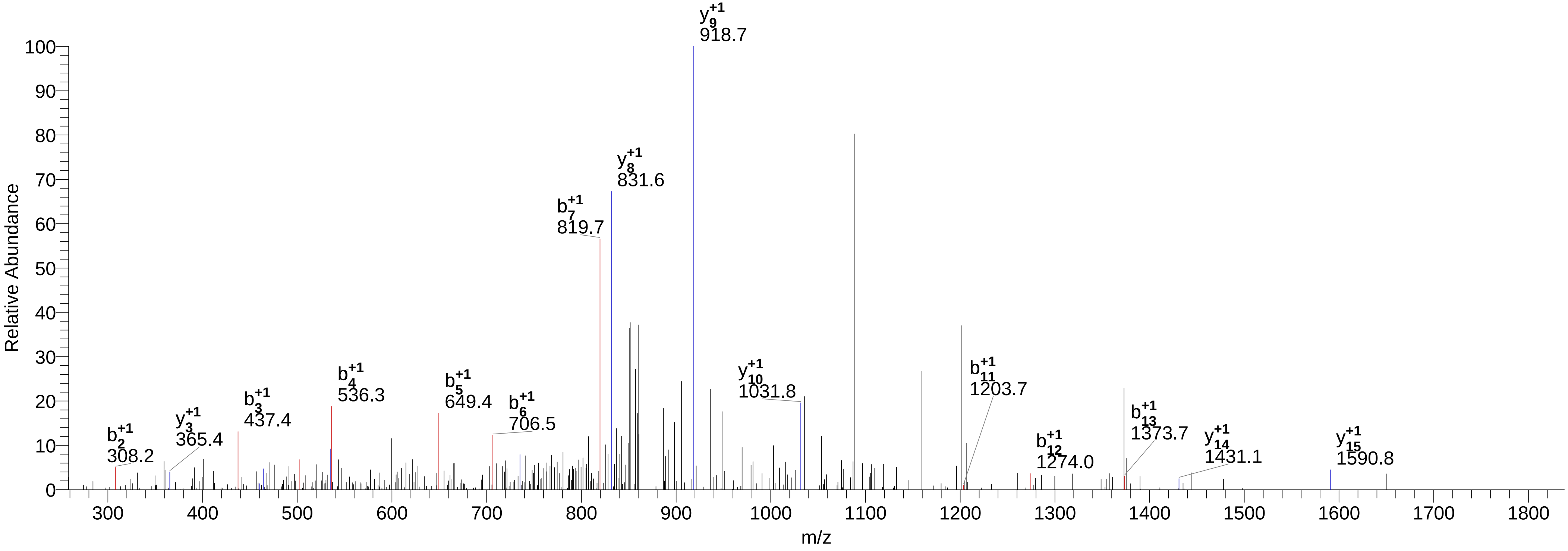

DTA: COIP_IAP.6549.6549.2
Precursor ion: 861.20
Mass type: Average
Mod's: (M* +15.9994) C=160.1652

Ion series for charge: +1

AA	A ions	B ions	B* ions	Bo ions	C ions	Y ions	Y* ions	Yo ions	Z ions
F		148.18							
C		**308.35**				1574.86			
E		**437.46**				**1414.70**			
V		**536.59**				**1285.58**			
L		**649.75**				1186.45			
G		**706.80**				**1073.29**			
L		**819.96**				**1016.24**			
S		907.04				**903.08**			
P		1004.15				**816.00**			
A		**1075.23**				718.89			
Q		1203.36				**647.81**			
A		1274.44				519.68			
V		1373.57				**448.60**			
A		**1444.65**				**349.47**			
M		**1575.85**				278.39			
K						147.20			

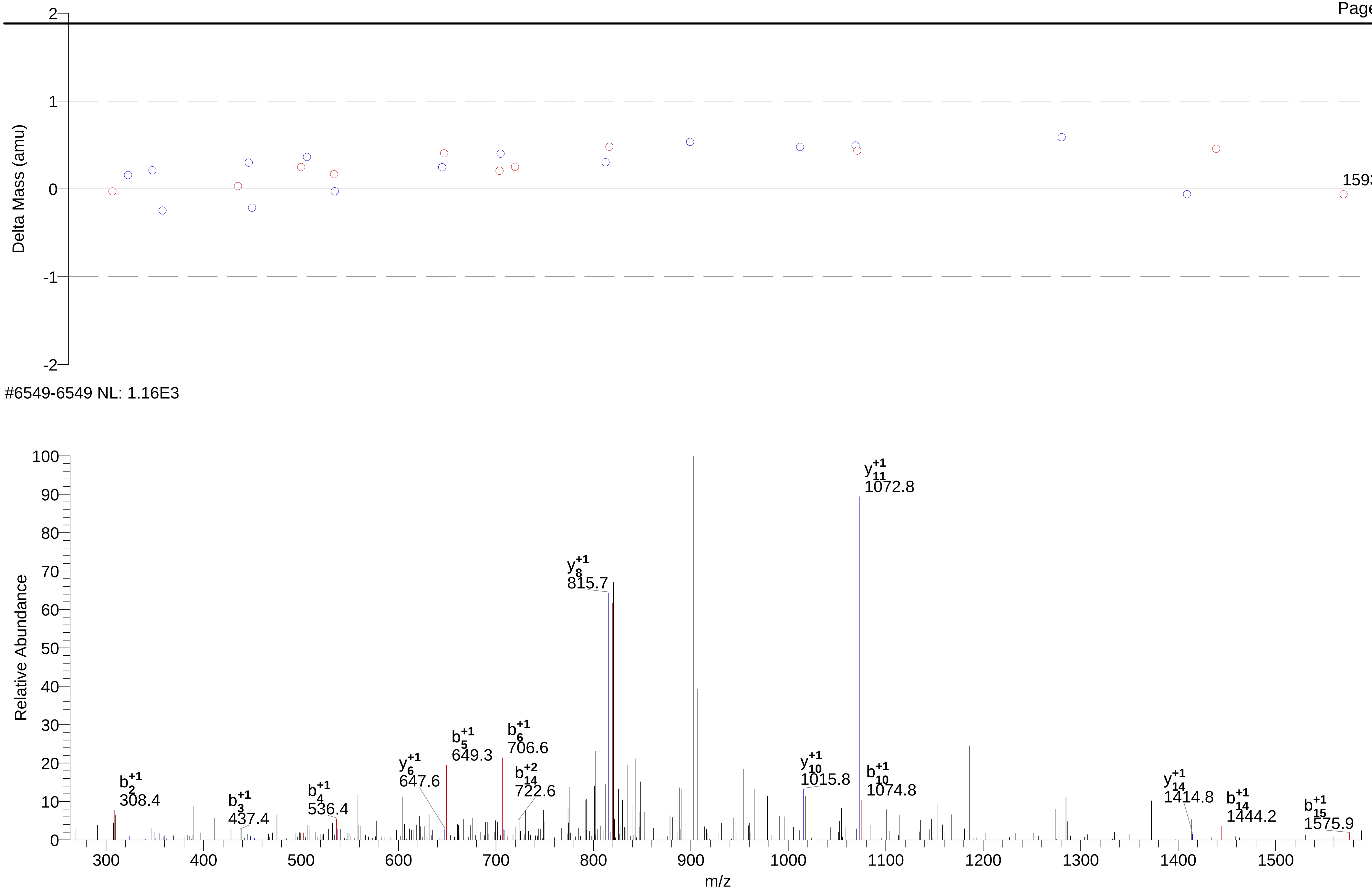
Delta Mass (amu)
2
1
0
-1
-2
1593
#6549-6549 NL: 1.16E3
Relative Abundance
m/z
y11 +1 1072.8
y8 +1 815.7
b2 +1 308.4
b3 +1 437.4
b4 +1 536.4
y6 +1 647.6
b5 +1 649.3
b6 +1 706.6
b14 +2 722.6
y10 +1 1015.8
b10 +1 1074.8
y14 +1 1414.8
b14 +1 1444.2
b15 +1 1575.9

DTA: COIP_IAP.6558.6558.2
Precursor ion: 861.43
Mass type: Average
Mod's: (M* +15.9994) C=160.1652

Ion series for charge: +1

AA	A ions	B ions	B* ions	Bo ions	C ions	Y ions	Y* ions	Yo ions	Z ions
F		148.18							
C		**308.35**				**1574.86**			
E		**437.46**				**1414.70**			
V		**536.59**				1285.58			
L		**649.75**				1186.45			
G		**706.80**				**1073.29**			
L		**819.96**				**1016.24**			
S		**907.04**				**903.08**			
P		1004.15				**816.00**			
A		1075.23				718.89			
Q		1203.36				647.81			
A		1274.44				**519.68**			
V		**1373.57**				**448.60**			
A		**1444.65**				**349.47**			
M		1575.85				**278.39**			
K						147.20			

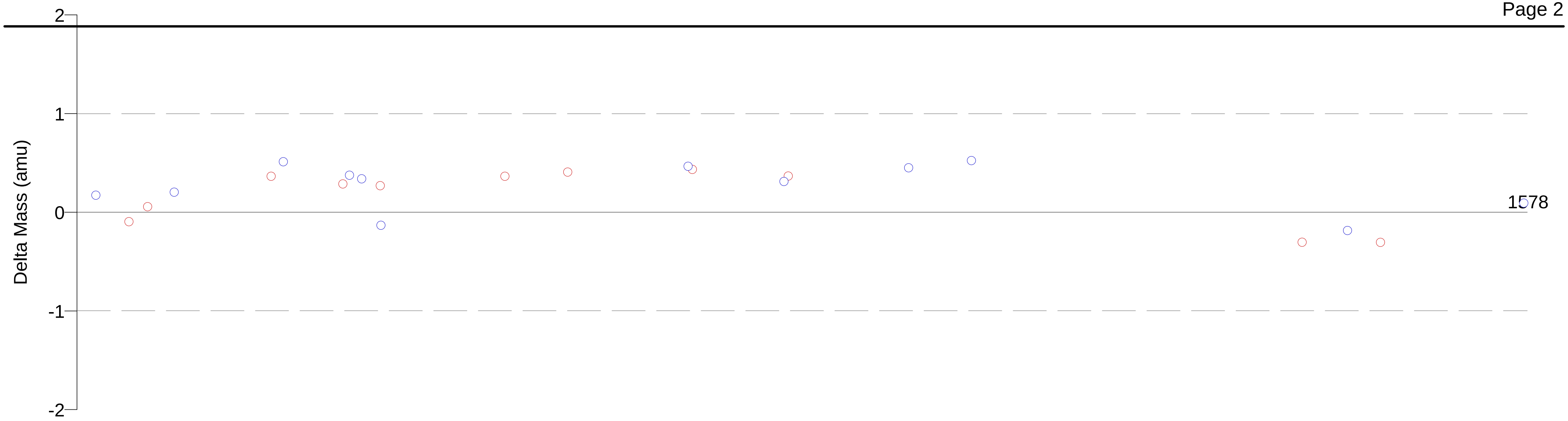

#6558-6558 NL: 1.43E3

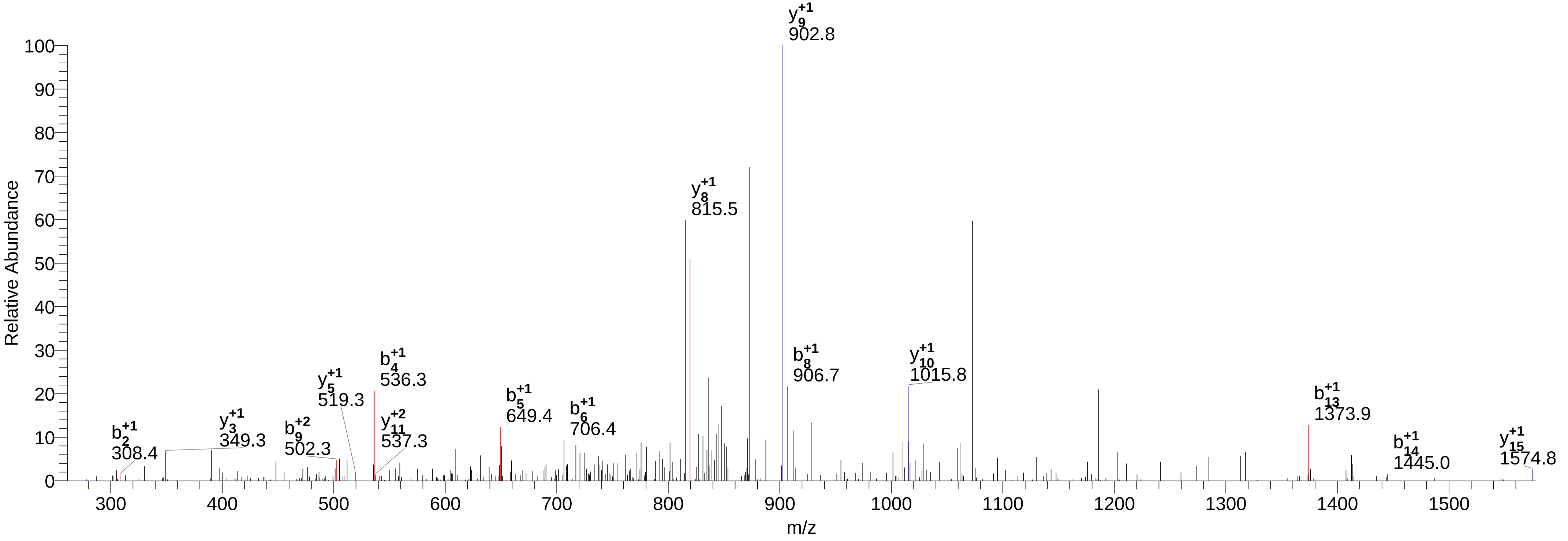

DTA: COIP_IAP.3913.3913.2
Precursor ion: 708.64
Mass type: Average
Mod's: (M* +15.9994) C=160.1652

Ion series for charge: +1

AA	A ions	B ions	B* ions	Bo ions	C ions	Y ions	Y* ions	Yo ions	Z ions
K		129.18							
L		**242.34**				1287.44			
D		**357.43**				1174.29			
E		**486.54**				**1059.20**			
K		**614.71**				**930.08**			
D		**729.80**				**801.91**			
Q		857.93				**686.82**			
A		929.01				558.69			
V		1028.14				**487.62**			
Q		1156.27				**388.48**			
L		**1269.43**				**260.35**			
K						147.20			

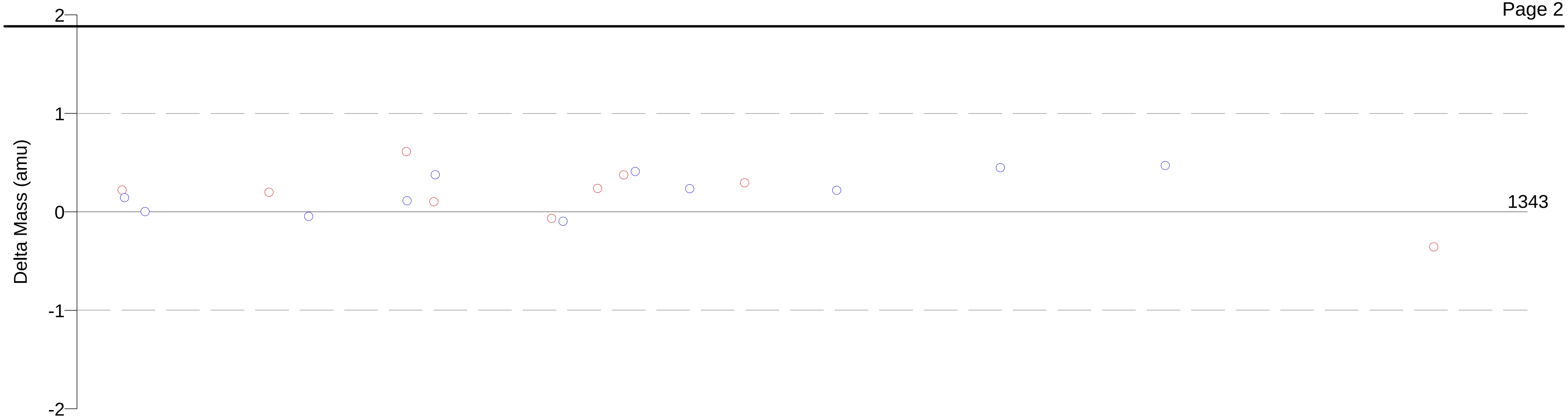

#3913-3913 NL: 1.23E3

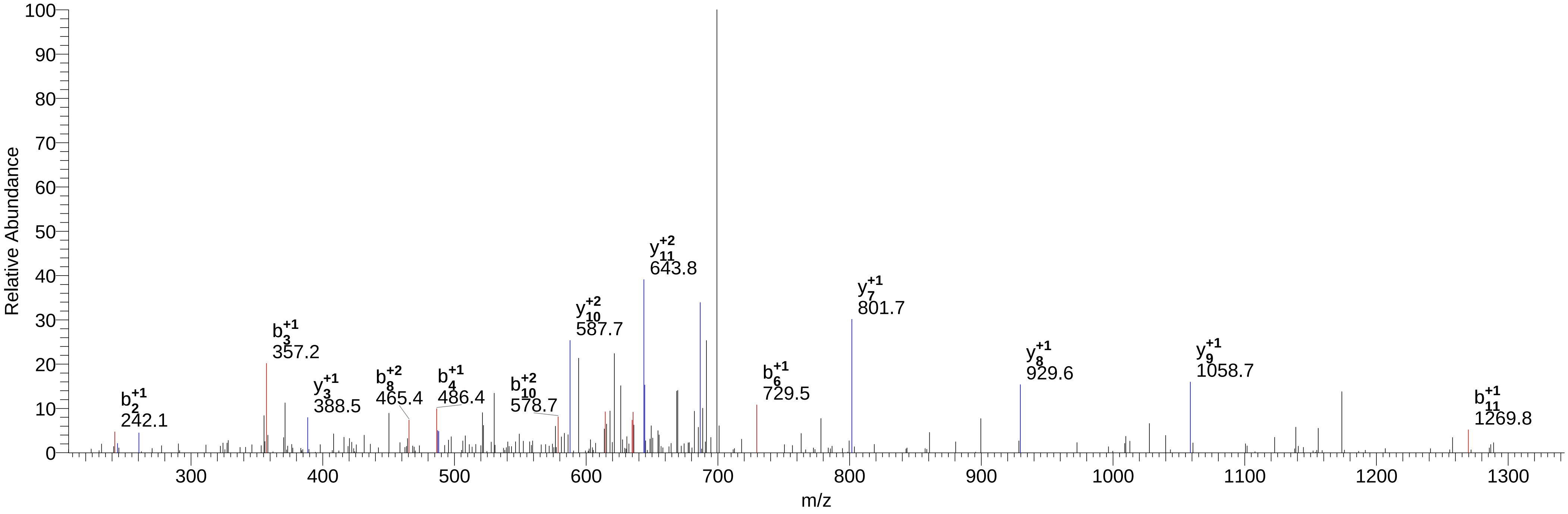

DTA: COIP_IAP.3954.3954.2
Precursor ion: 708.31
Mass type: Average
Mod's: (M* +15.9994) C=160.1652

Ion series for charge: +1

AA	A ions	B ions	B* ions	Bo ions	C ions	Y ions	Y* ions	Yo ions	Z ions
K		129.18							
L		**242.34**				1287.44			
D		**357.43**				1174.29			
E		486.54				**1059.20**			
K		**614.71**				**930.08**			
D		**729.80**				**801.91**			
Q		**857.93**				**686.82**			
A		929.01				558.69			
V		**1028.14**				**487.62**			
Q		**1156.27**				**388.48**			
L		1269.43				**260.35**			
K						147.20			

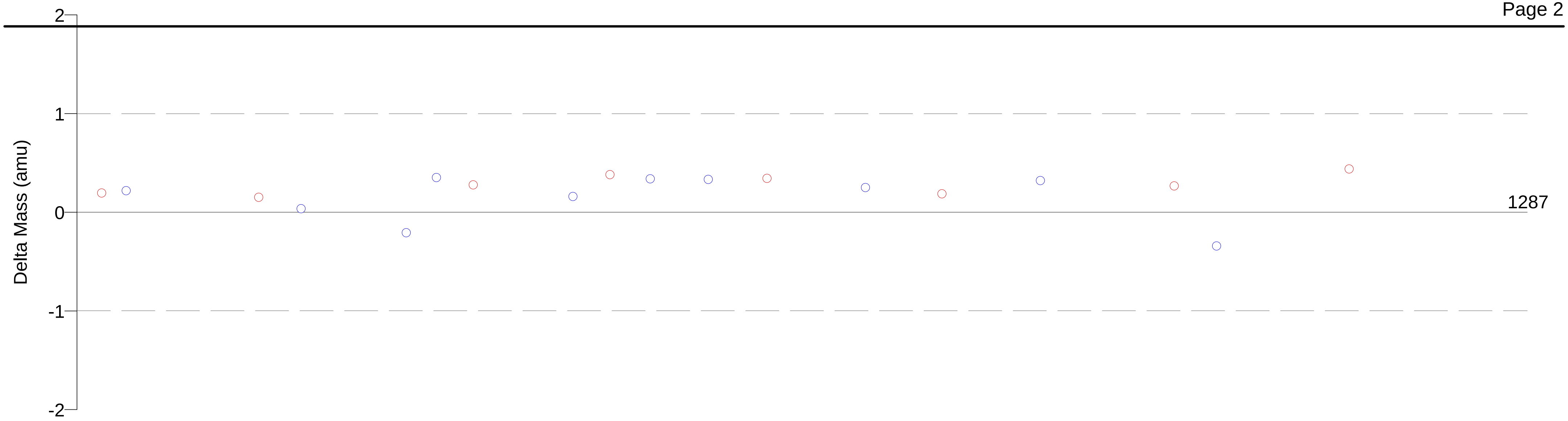

#3954-3954 NL: 4.53E2

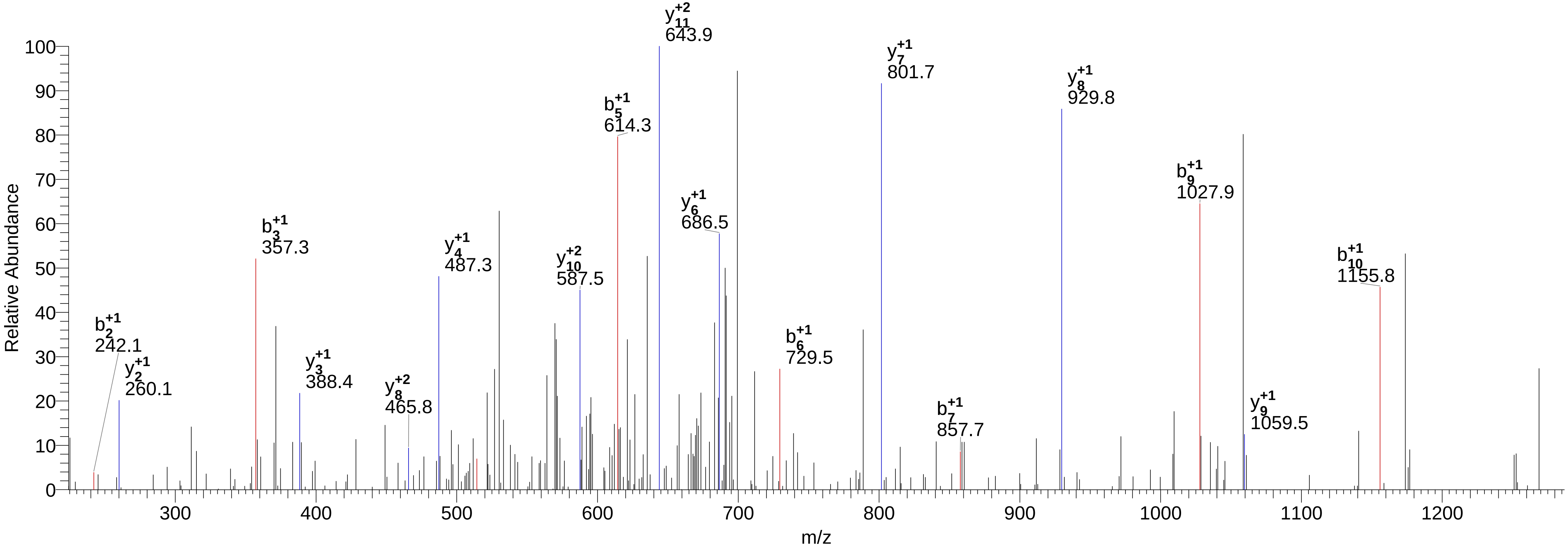

DTA: COIP_IAP.6498.6498.2
Precursor ion: 940.93
Mass type: Average
Mod's: (M* +15.9994) C=160.1652

Ion series for charge: +1

AA	A ions	B ions	B* ions	Bo ions	C ions	Y ions	Y* ions	Yo ions	Z ions
L		114.17							
H		251.31				1768.00			
P		348.42				1630.86			
D		**463.51**				**1533.75**			
V		562.64				1418.66			
T		**663.74**				1319.53			
V		**762.88**				**1218.42**			
I		**876.03**				**1119.29**			
Y		**1039.21**				**1006.13**			
E		1168.32				842.96			
Q		**1296.45**				**713.85**			
L		1409.61				**585.72**			
P		**1506.73**				**472.56**			
L		1619.89				375.44			
D		**1734.97**				262.28			
K						147.20			

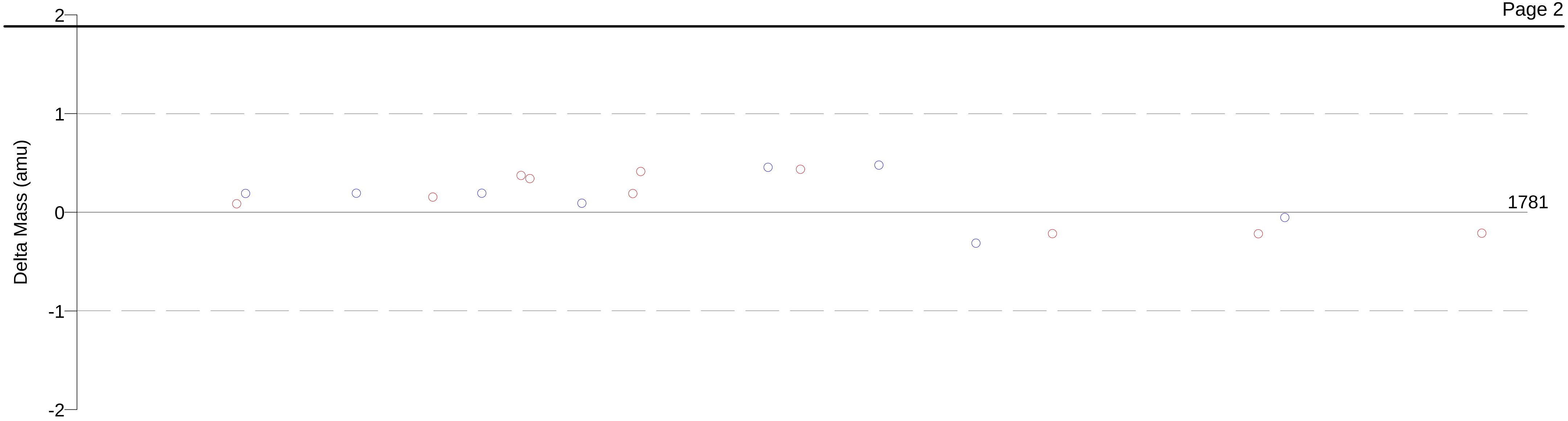

#6498-6498 NL: 1.78E3

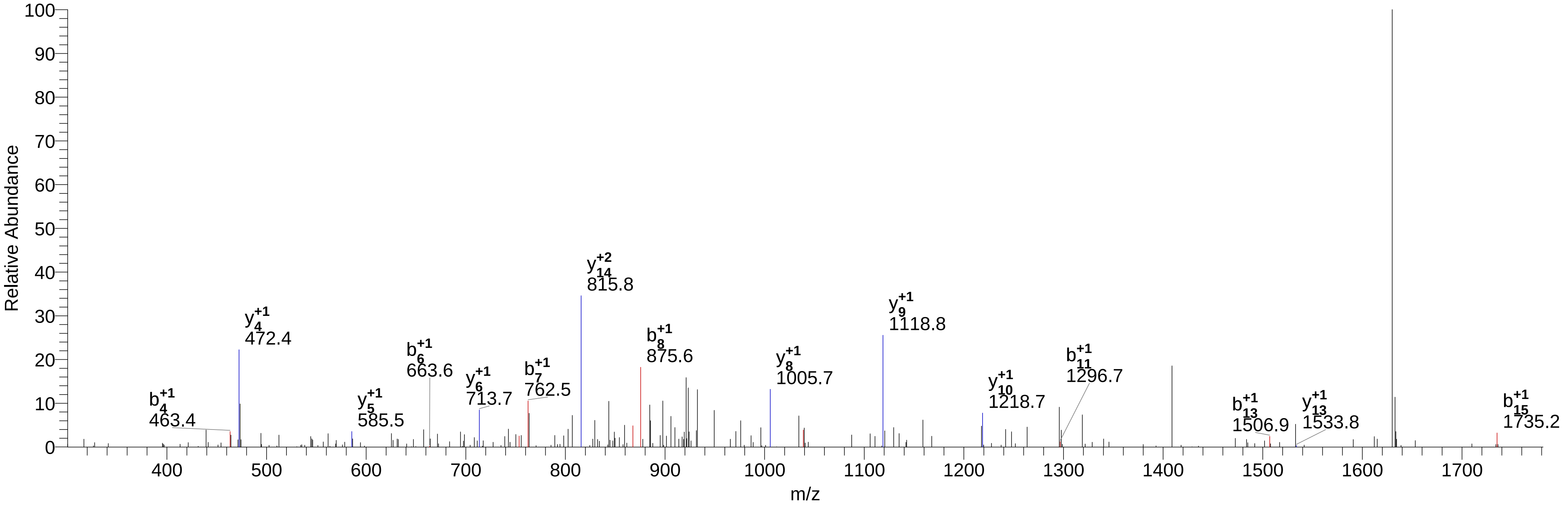

DTA: COIP_IAP.6540.6540.2
Precursor ion: 940.91
Mass type: Average
Mod's: (M* +15.9994) C=160.1652

Ion series for charge: +1

AA	A ions	B ions	B* ions	Bo ions	C ions	Y ions	Y* ions	Yo ions	Z ions
L		114.17							
H		251.31				1768.00			
P		348.42				1630.86			
D		**463.51**				1533.75			
V		**562.64**				**1418.66**			
T		**663.74**				1319.53			
V		**762.88**				1218.42			
I		**876.03**				1119.29			
Y		**1039.21**				**1006.13**			
E		**1168.32**				**842.96**			
Q		1296.45				**713.85**			
L		**1409.61**				**585.72**			
P		1506.73				**472.56**			
L		**1619.89**				375.44			
D		1734.97				262.28			
K						147.20			

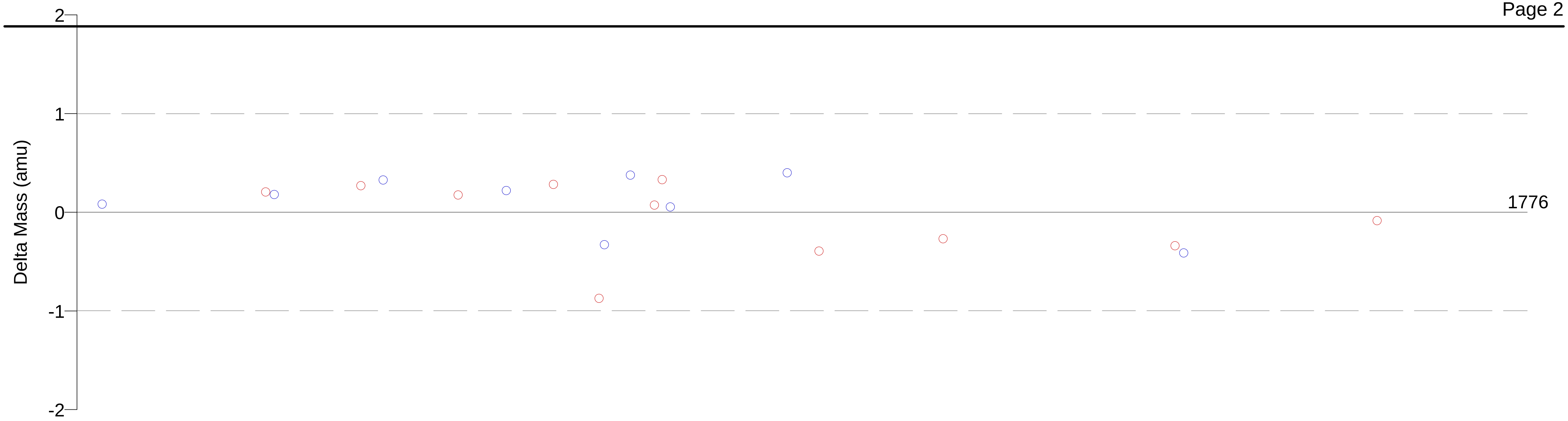

#6540-6540 NL: 2.43E3

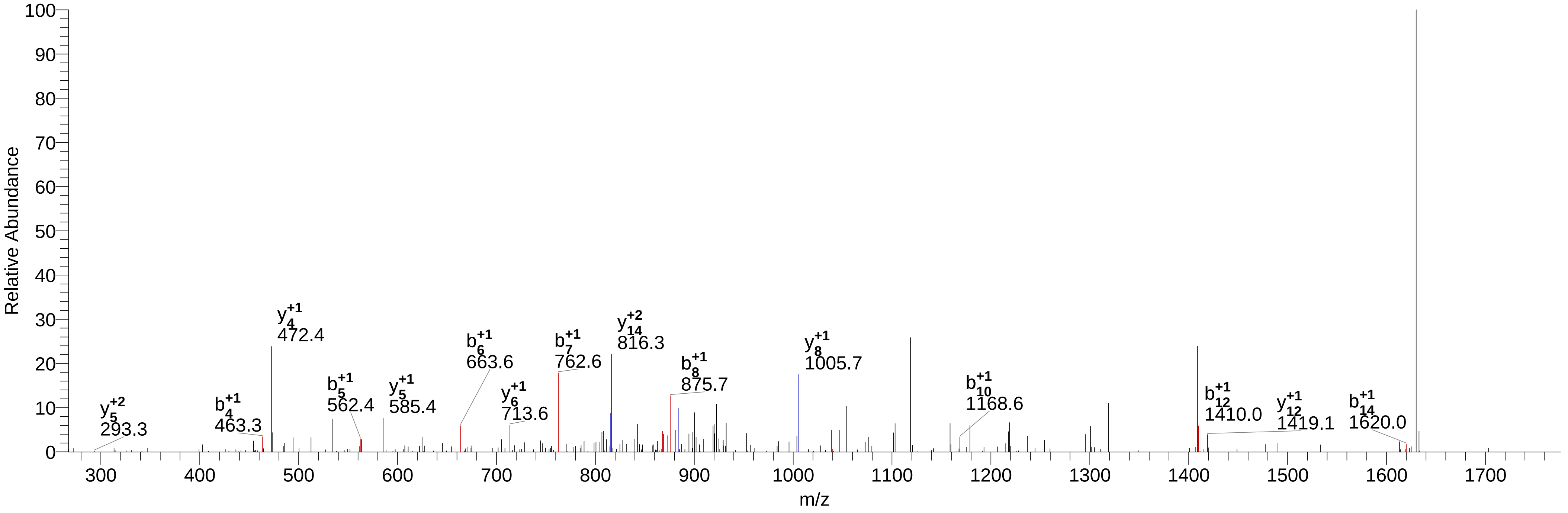

DTA: COIP_IAP.6682.6682.3
Precursor ion: 627.59
Mass type: Average
Mod's: (M* +15.9994) C=160.1652

Ion series for charge: +1

AA	A ions	B ions	B* ions	Bo ions	C ions	Y ions	Y* ions	Yo ions	Z ions
L		114.17							
H		**251.31**				1768.00			
P		**348.42**				1630.86			
D		**463.51**				1533.75			
V		562.64				1418.66			
T		**663.74**				1319.53			
V		**762.88**				1218.42			
I		**876.03**				**1119.29**			
Y		**1039.21**				**1006.13**			
E		1168.32				**842.96**			
Q		**1296.45**				**713.85**			
L		1409.61				**585.72**			
P		1506.73				**472.56**			
L		1619.89				375.44			
D		1734.97				**262.28**			
K						147.20			

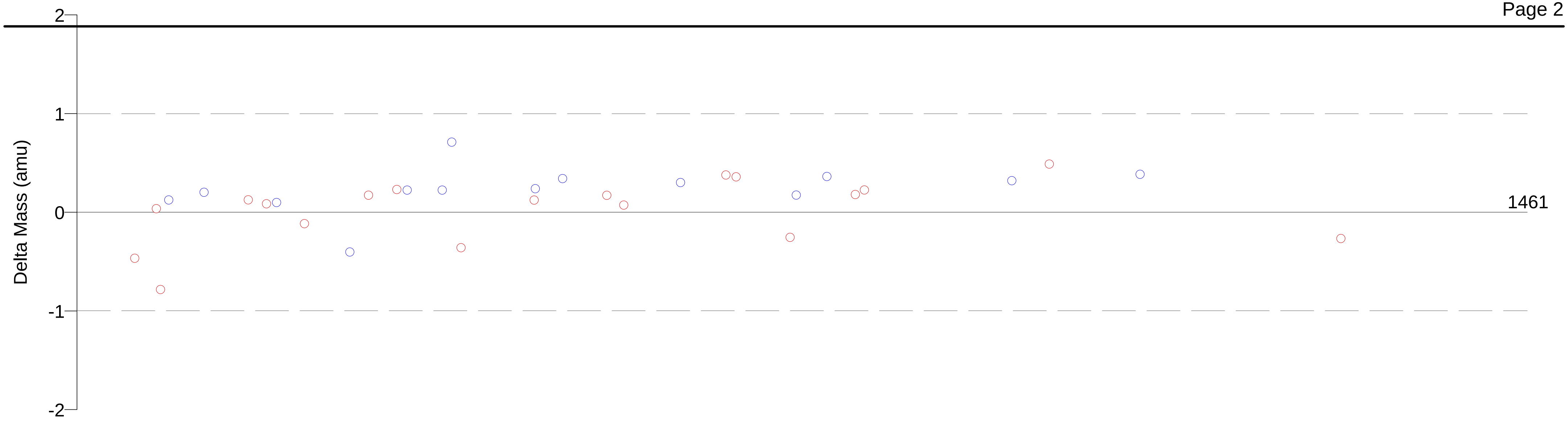

#6682-6682 NL: 4.87E3

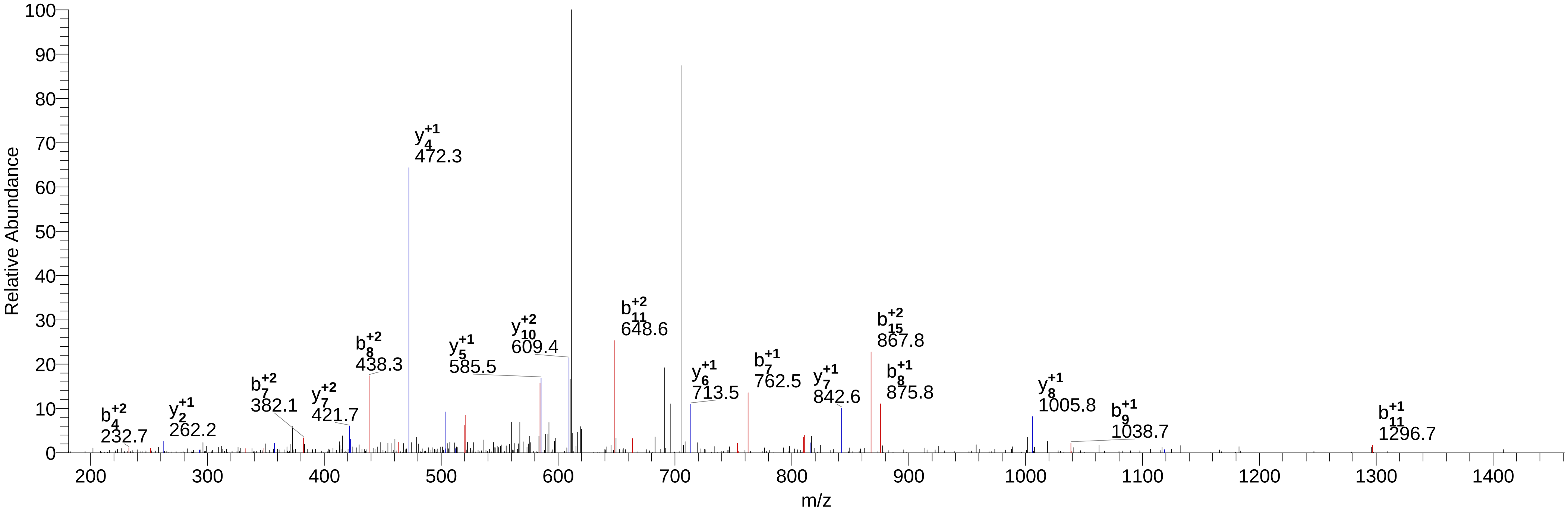

DTA: COIP_IAP.3588.3588.1
Precursor ion: 773.58
Mass type: Average
Mod's: (M* +15.9994) C=160.1652

Ion series for charge: +1

AA	A ions	B ions	B* ions	Bo ions	C ions	Y ions	Y* ions	Yo ions	Z ions
Q		129.14							
I		242.30				645.77			
A		**313.37**				**532.61**			
I		**426.53**				**461.53**			
S		**513.61**				**348.38**			
N		**627.71**				**261.30**			
K						147.20			

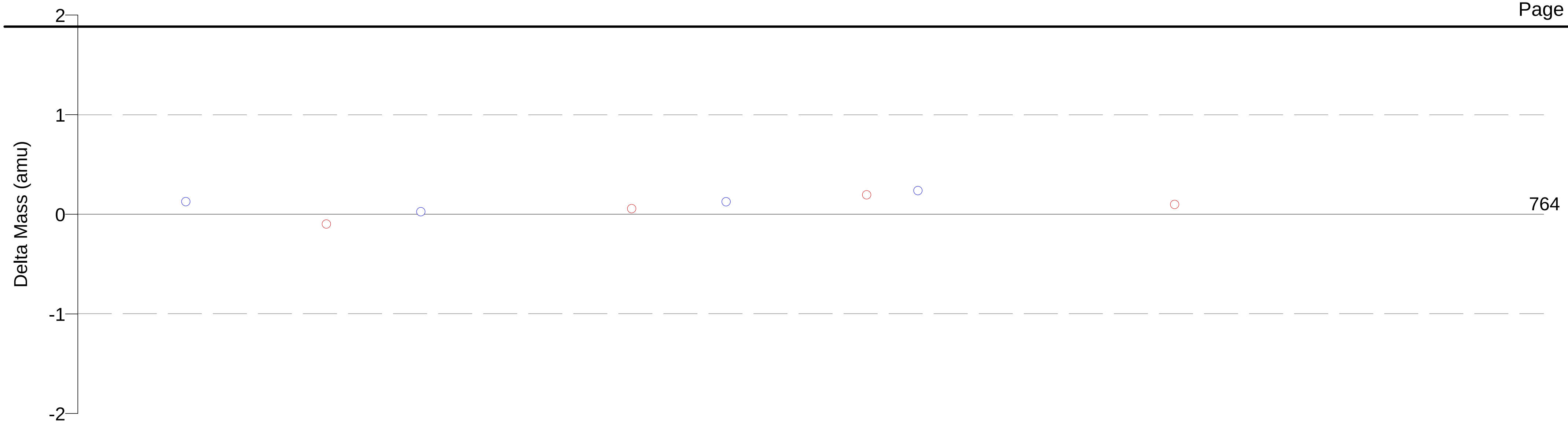

#3588-3588 NL: 3.77E3

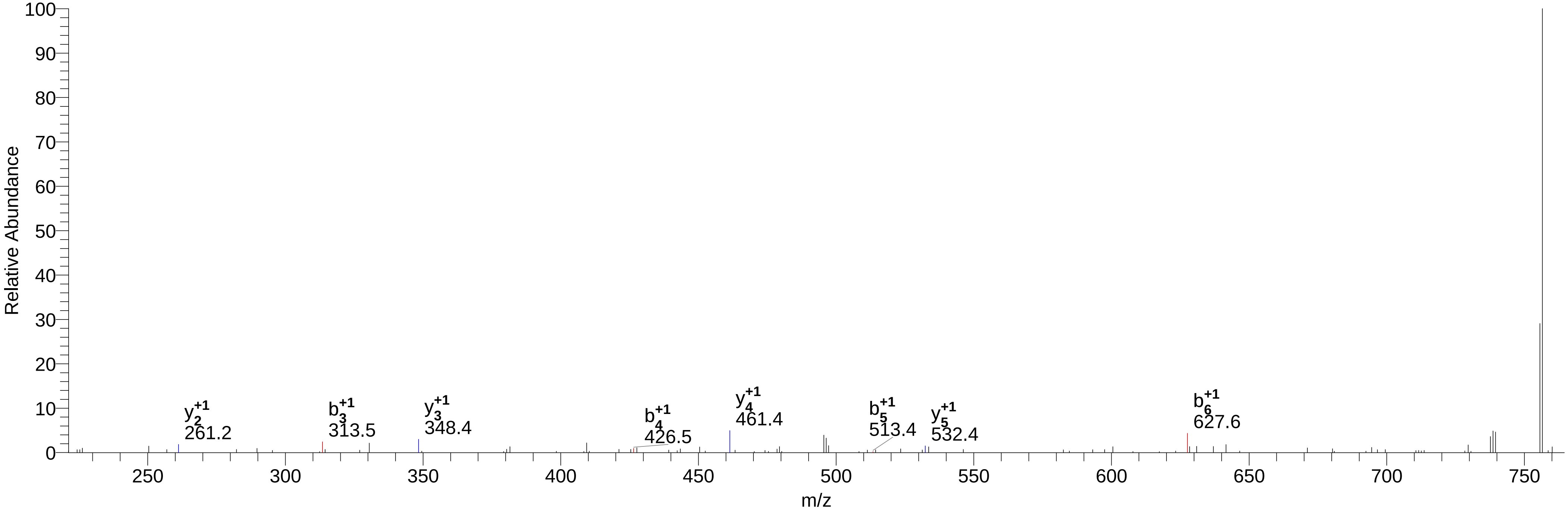

DTA: COIP_IAP.6140.6140.2
Precursor ion: 589.32
Mass type: Average
Mod's: (M* +15.9994) C=160.1652

Ion series for charge: +1

AA	A ions	B ions	B* ions	Bo ions	C ions	Y ions	Y* ions	Yo ions	Z ions
W		**187.22**							
Q		**315.35**				991.08			
T		**416.45**				**862.95**			
L		529.61				**761.85**			
F		**676.79**				**648.69**			
D		**791.87**				**501.52**			
P		888.99				**386.43**			
N		1003.09				289.31			
R						**175.21**			

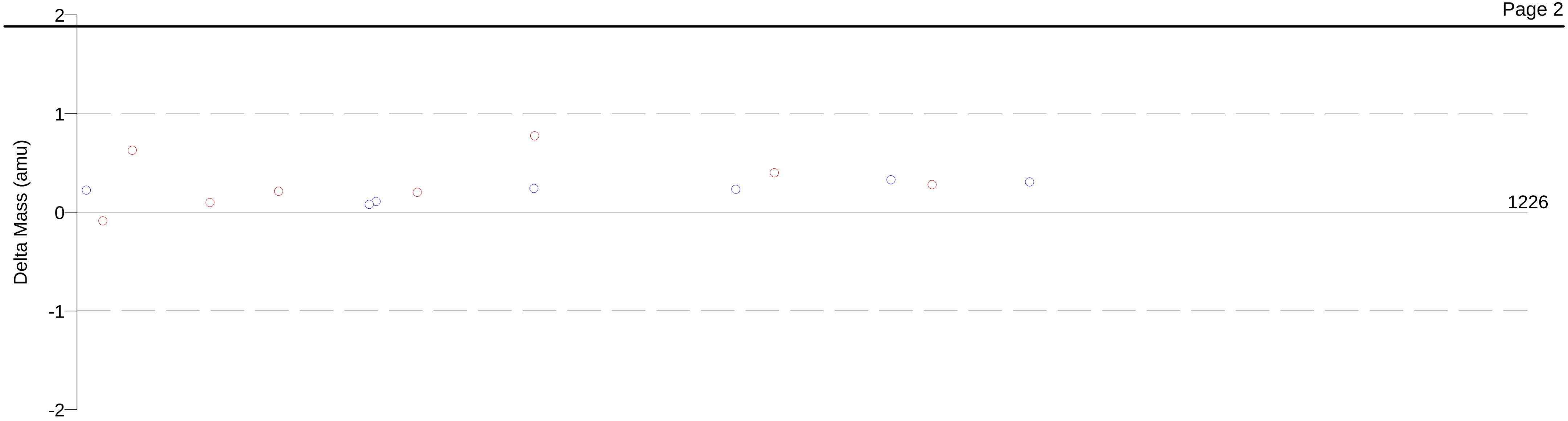

#6140-6140 NL: 3.44E3

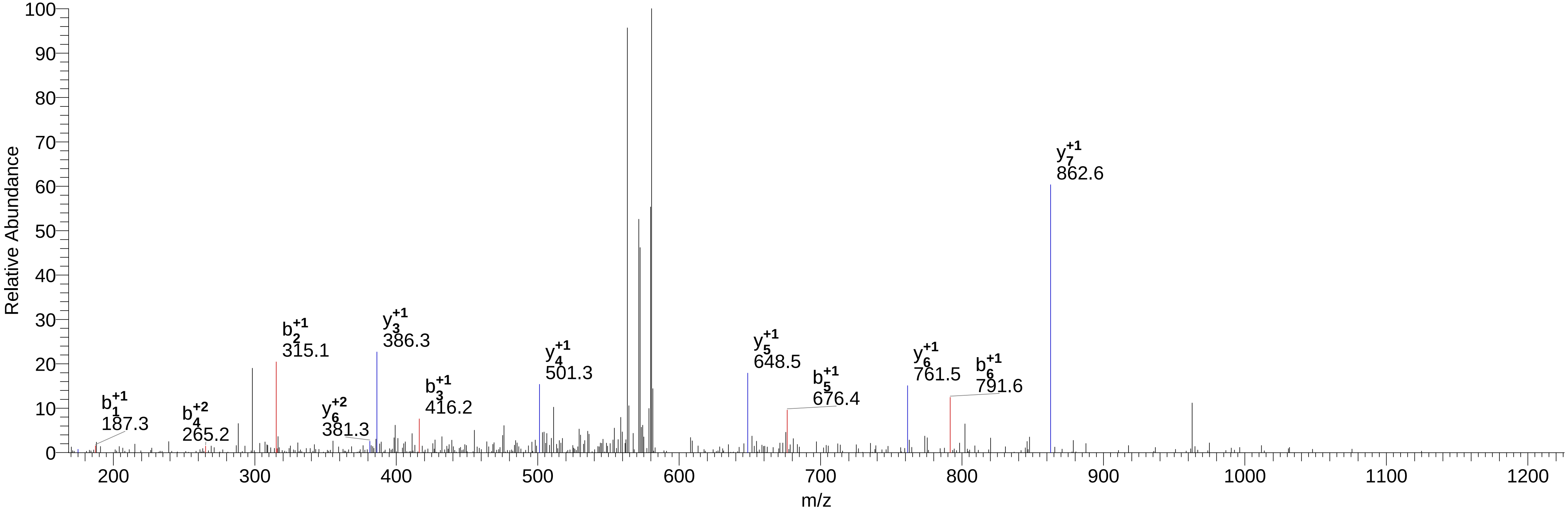

DTA: COIP_IAP.5229.5229.2
Precursor ion: 729.53
Mass type: Average
Mod's: (M* +15.9994) C=160.1652

Ion series for charge: +1

AA	A ions	B ions	B* ions	Bo ions	C ions	Y ions	Y* ions	Yo ions	Z ions
G		58.06							
S		145.14				1401.46			
F		**292.31**				1314.39			
W		**478.52**				**1167.21**			
S		**565.60**				**981.00**			
S		652.68				**893.92**			
Y		815.85				**806.85**			
S		**902.93**				**643.67**			
H		**1040.07**				**556.59**			
S		1127.15				**419.45**			
A		1198.23				**332.38**			
N		**1312.33**				**261.30**			
K						147.20			

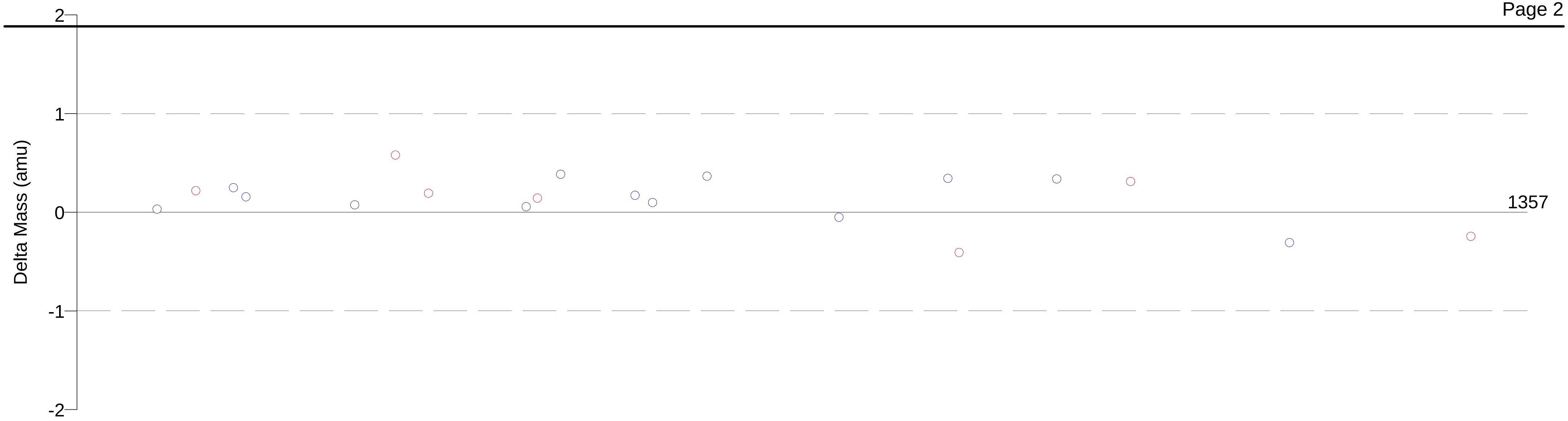

#5229-5229 NL: 1.01E3

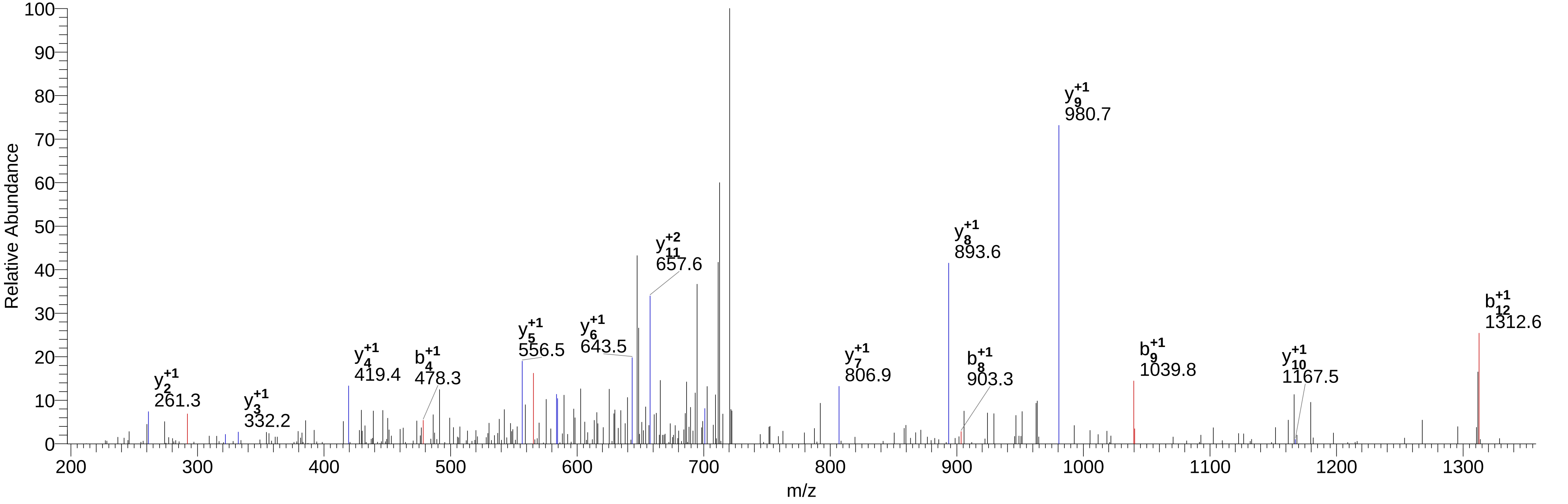

Supplement: S1 Fig — (PDF) [file pntd.0006654.s001.pdf]
