# Supplementary figures and images for: Schistosoma japonicum IAP and Teg20 safeguard tegumental integrity by inhibiting cellular apoptosis
Source: PLoS Negl Trop Dis. 2018 Jul 25;12(7):e0006654. doi: 10.1371/journal.pntd.0006654 (PMC6078320; doi:10.1371/journal.pntd.0006654)

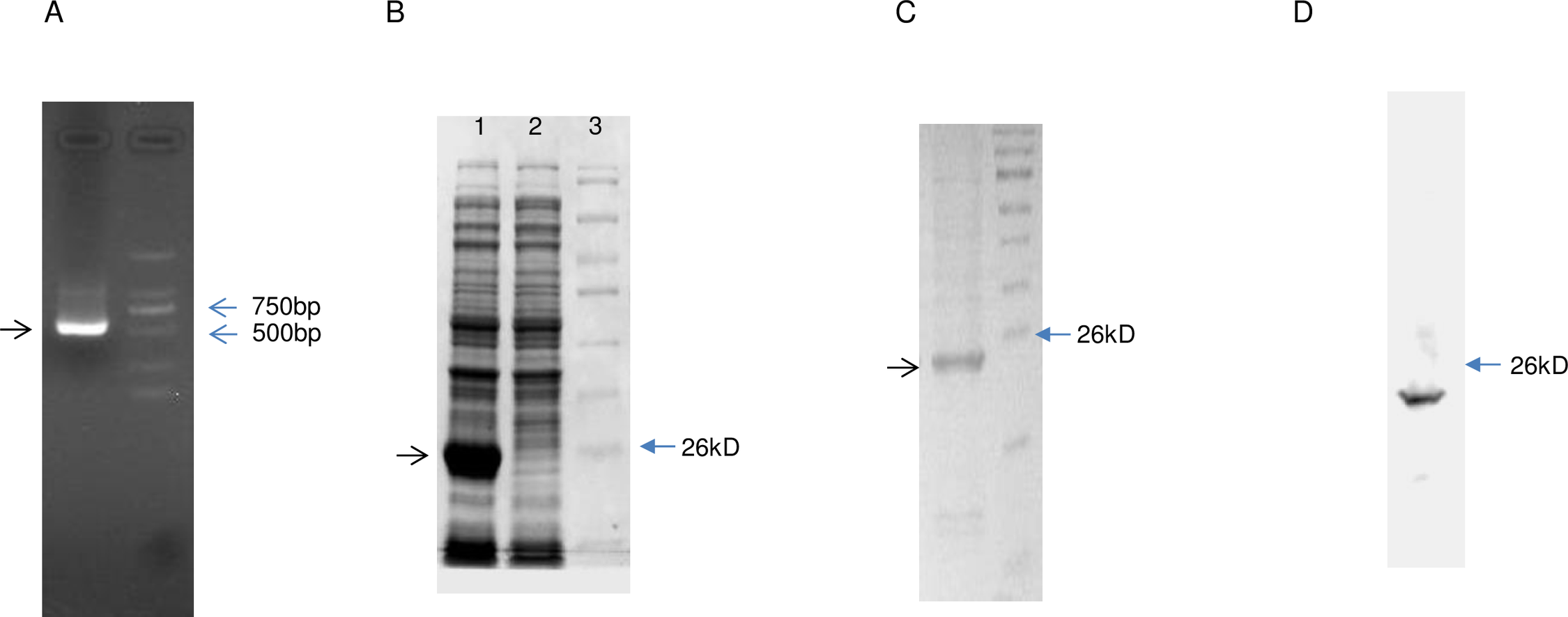

Supplement: S2 Fig — (A) Agarose gel analysis of PCR product for amplifying SjTeg-20. (B) SDS-PAGE analysis of the expression of recombinant SjTeg-20 in E. coli. 1. E. coli transfected with recombinant plasmid encoding SjTeg-20; 2. E. coli transfected with control plasmid; 3. Protein marker. (C) SDS-PAGE analysis of purified recombinant SjTeg-20. (D) Western blot analysis of sera against recombinant SjTeg-20. The arrow indicates the target product. (TIF) [file pntd.0006654.s002.tif]
