# Supplementary material for: Schistosoma japonicum IAP and Teg20 safeguard tegumental integrity by inhibiting cellular apoptosis
Source: PLoS Negl Trop Dis. 2018 Jul 25;12(7):e0006654. doi: 10.1371/journal.pntd.0006654 (PMC6078320; doi:10.1371/journal.pntd.0006654)
Supplement: S1 Table — (DOCX) [file pntd.0006654.s003.docx]

S Table 1 List of siRNA used for silencing

| Gene IDs | siRNA names | Sequences(5`---3`) |
| --- | --- | --- |
| IAP | siRNA-951 | Sense CCG CCU ACA AAG UCA AUC UTT  Antisense AGA UUG ACU UUG UAG GCG GTT |
| Teg20 | siRNA-132 | Sense GGCUGCAAAUAAUCUUGAUTT  Antisense AUCAAGAUUAUUUGCAGCCTT |
|  | siRNA-621 | Sense GCAAGCUUGGCGUAAUCAUTT  Antisense AUGAUUACGCCAAGCUUGCTT |
|  | siRNA-802 | Sense GCCAGCUGCAUUAAUGAAUTT  Antisense AUUCAUUAAUGCAGCUGGCTT |
| Control siRNA |  | Sense UGG CGA AUG GCC GGA CAC UCC TT  Antisense GGA GUG UCC GGC CAU UCG CAA TT |
